# Structural basis of terephthalate recognition by solute binding protein TphC

**DOI:** 10.1038/s41467-021-26508-0

**Published:** 2021-10-29

**Authors:** Trishnamoni Gautom, Dharmendra Dheeman, Colin Levy, Thomas Butterfield, Guadalupe Alvarez Gonzalez, Philip Le Roy, Lewis Caiger, Karl Fisher, Linus Johannissen, Neil Dixon

**Affiliations:** 1 Manchester Institute of Biotechnology (MIB) and Department of Chemistry, The University of Manchester, Manchester, UK; 2 Department of Biotechnology, Gauhati University, Guwahati, Assam India; 3 Present Address: Royal School of Bio-Sciences, Royal Global University, Guwahati, Assam India

**Keywords:** Transporters, Biophysical chemistry

## Abstract

Biological degradation of Polyethylene terephthalate (PET) plastic and assimilation of the corresponding monomers ethylene glycol and terephthalate (TPA) into central metabolism offers an attractive route for bio-based molecular recycling and bioremediation applications. A key step is the cellular uptake of the non-permeable TPA into bacterial cells which has been shown to be dependent upon the presence of the key *tphC* gene. However, little is known from a biochemical and structural perspective about the encoded solute binding protein, TphC. Here, we report the biochemical and structural characterisation of TphC in both open and TPA-bound closed conformations. This analysis demonstrates the narrow ligand specificity of TphC towards aromatic para-substituted dicarboxylates, such as TPA and closely related analogues. Further phylogenetic and genomic context analysis of the *tph* genes reveals homologous operons as a genetic resource for future biotechnological and metabolic engineering efforts towards circular plastic bio-economy solutions.

## Abstract (web summary)

The presence of the gene encoding the solute binding protein TphC has been shown to permit the uptake of terephthalate (TPA), which is the breakdown product of Polyethylene terephthalate (PET) plastic. Here the authors present a structural characterization of TphC in both open and TPA-bound closed conformations.

## Introduction

The global appetite for plastic products has transitioned our planet into an era of the “Plastic Age”^1^. Polyethylene terephthalate (PET) plastic is the most commonly used plastic in the packaging of beverages, food and pharmaceuticals. Since its first synthesis back in 1941, PET has gradually emerged as the world’s favorite food-safe plastic due to its robustness, chemical inertness and durability. Although regarded as non-toxic and 100% recyclable, single-use convenience-sized PET bottles have made PET plastic the third most collected debris in beach clean-ups in more than 100 countries^2^ and is overwhelmingly omnipresent in the terrestrial ecosystem^3,4^. The post-consumer recyclability of plastics is still questionable owing to a number of factors^5,6^ that have turned PET from a miraculous material into the scourge of the land and sea. For instance, the PET-recycling rate was estimated to be only ~30% in 2015 in the US, and in Europe 25% of post-consumer plastic waste still went into landfill in 2018 (ref. ^7^). This evidently ubiquitous plastic footprint, however, has not impacted upon the demand and production given the essentiality of plastics in our daily lives. The global PET market in 2017 was valued at ~USD 24 billion and is expected to hit USD 39 billion by 2027 (ref. ^8^). In 2016, around 485 billion PET bottles were manufactured, and around 583.3 billion are forecast to be produced in 2021 (ref. ^9^). The global demand for PET in 2030 is forecast to amount to 42 metric tons^10^.

**Fig. 1:**
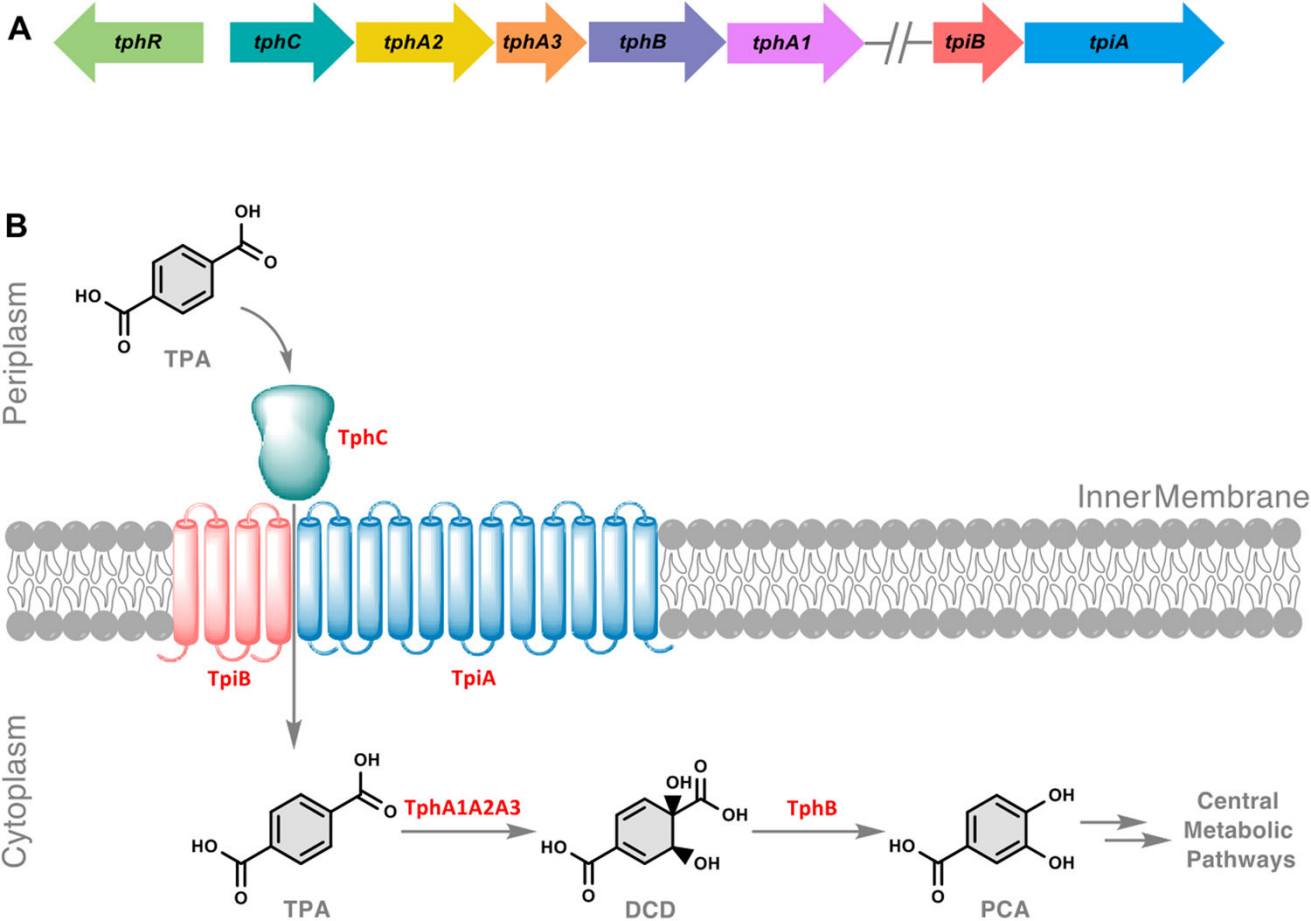
Terephthalate cellular uptake and assimilation operons. **A** Schematic representation of *tph* and *tpi* operons in the chromosome of *Comamonas* sp. strain E6. The catabolic genes of the *tph* operon, encoding a three-component TPA 1,2-dioxygenase (TPADO), are represented in gold (*tphA2*), orange (*tphA3*) and pink (*tphA*1). The *tph* operon regulator (*tphR*), a gene encoding a diol dehydrogenase (*tphB*) and a gene encoding a periplasmic solute-binding protein (*tphC*), are represented in green, teal and mauve, respectively. The transport genes (*tpiBA*) in *tpi* operon, encoding a set of transmembrane transport proteins, are shown in peach and light blue, respectively. **B** Schematic of TPA transport and catabolism. The *tphR* encoded activator (TphR) responds to the presence of inducer TPA and leads to the expression of a set of capture, transport and catabolic proteins to metabolise TPA in a successive binding, transport and catabolic steps: TphC in the periplasm first binds to TPA and relays it to a pair of transmembrane proteins, TpiB and TpiA, which in turn transport it to the cytoplasm, where it is converted by a three-component TPADO to 1,6-dihydroxycyclohexa-2,4-diene-dicarboxylate (DCD). The DCD is acted upon by TphB (1,2-dihydroxy-3,5-cyclohexadiene-1,4-dicarboxylate dehydrogenase) and converted to protocatechuate (PCA), which is then funnelled into the central metabolic pathways.

Both mechanical and chemical PET-recycling methods are available; however, widespread use is limited^11^. Indeed, some concerns remain over the product quality and environment impact of the mechanical and chemical PET recycling, respectively^6^. Microbial and enzyme-based plastic waste biodegradation and recycling offer promising alternative strategies due to the use of benign conditions and potential as a cost-effective environment-friendly approach^12–16^. Therefore, there is great interest in finding better strategies for PET bioconversion and recycling through engineering robust enzymes and microbial strains for its degradation, uptake and assimilation. Terephthalic acid (TPA) and ethylene glycol (EG), which together form a polymer chain, are the basic building blocks of PET and can be released by enzymatic hydrolysis via the action of different types of bacterial and fungal origin hydrolases, such as esterases, lipases, cutinases and carboxylesterases^17–24^. A bacterial strain *Ideonella sakaiensis* was discovered that secreted two enzymes PETase and MHETase which enable the microbe to grow on low crystalline PET film as its major carbon source under optimised non-native lab conditions^25^. PET is initially hydrolysed to monohydroxyethyl terephthalate (MHET) by PETase which is subsequently hydrolysed into TPA and EG by MHETase. Following their discovery, these two enzymes have been extensively studied biochemically and structurally, and further subjected to directed evolution to enhance their catalytic activities and substrate specificities^26–32^. The constituent monomers, EG and TPA, can then be taken up and degraded by microorganisms competent to utilise these compounds for their metabolism. For instance, *Pseudomonas putida* is capable of directly funnelling EG to the Krebs cycle via isocitrate^33^. Similarly, TPA can be converted to protocatechuic acid (PCA) via an initial dioxygenolytic step in which the aromatic ring is cleaved before entering the central metabolism^34,35^. TPA is converted to PCA via a pathway encoded by the *tph* operon (Fig. 1A), these genes have been characterised in the β-proteobacteria *Comamonas testosteroni* YZW-D^36^, *Comamonas* sp. strain E6 (refs. ^37,38^) and in the actinomycete *Rhodococcus* sp. strain DK17 (refs. ^39^).

To date, unlike the enzymes involved in the degradation of PET and assimilation of the breakdown products, the cellular uptake of these monomers, EG and TPA, has received limited attention. Adaptive laboratory evolution was used to engineer *Pseudomonas putida* KT2440 to enhance EG cellular uptake and metabolism^40,41^, and heterologous expression of the tph pathway has been reported in a number of different hosts^13,37,42–44^. Cellular uptake of TPA into *Comamonas* sp. strain E6 has been shown to be dependent upon the presence of the *tphC* gene and its product TphC^37,45^, which is predicted to be a solute-binding protein (SBP) belonging to the tripartite tricarboxylate transporters (TTT) class of transporters^46^. The initial ligand recognition in a TTT system is performed by the periplasmic SBP (Fig. 1B). TTT-SBPs have also been identified in uptake mechanisms for other biotechnologically important ligands, such as C_6_ dicarboxylic acids^47^, C_4_ dicarboxylic acids^48^, sulfolactate^49^ and the synthetic precursors for polythioesters^50^. Apart from Bug27 (ref. ^51^), the *Bordetella pertussis* SBP for nicotinic acid and related compounds, TTT-SBPs have been shown to bind exclusively to dicarboxylic acids. This prokaryotic secondary solute transporter family is scarcely characterised, the ligand specificity of only a few TTT-SBP systems is known and consequently the SBP–ligand interactions are poorly understood. So far, the structure of only six TTT-SBPs have been determined, two from *Rhodopseudomonas palustris*^47,48^, three from *Bordetella pertussis*^51–53^ and one from *Polaromonas* sp. These protein structures show a common “Venus flytrap” fold comprising of two globular domains separated by a cleft that folds around the ligand. Despite cellular uptake of TPA being a key step, the confirmation of *tphC-*dependent uptake of TPA at the genetic-level^45^, and the potential for TphC to be used in engineered strains for the cellular uptake of TPA and bioconversion of plastic waste^14^, there have been no reports to date on the biochemical or structural characterisation of TphC. Due to the relatively recent xenobiotic introduction of PET and TPA into the environment, we were inspired to explore whether TPA is indeed the cognate ligand for TphC, or whether other closely related chemicals naturally found in the environment are also recognised by TphC.

Here, we employed differential scanning fluorescence (DSF) assay, mutational analysis, isothermal titration calorimetry (ITC) and X-ray crystallography to biochemically and structurally characterise TphC from *Comamonas* sp. strain E6. Further phylogenetic and taxonomic analysis was used to reveal homologous operons to explore the diversity and origin of these xenobiotic catabolism genes and to provide a genetic resource for future biotechnological and metabolic engineering efforts towards circular plastic bio-economy solutions.

## Results

### Production and purification of TphC

The pre-protein form of TphC from *Comamonas* sp. strain E6 contains a predicted signal peptide sequence and the mature form is composed of 294 amino acids (N29-L322) (Supplementary Fig. 1). In order to characterise TphC, the recombinant gene, encoding the mature form, was cloned and then overexpressed in *Escherichia coli* BL21(DE3), and the protein was purified in two consecutive chromatographic steps of metal-affinity chromatography and gel-filtration chromatography. Sodium dodecyl sulfate polyacrylamide gel electrophoresis (SDS-PAGE) analysis of the eluates shows the presence of an expected ~34 kDa protein band equivalent to an N-terminal 6×His-tag fusion of TphC (Supplementary Fig. 1).

### Ligand specificity screening with differential scanning fluorimetry

**Fig. 2:**
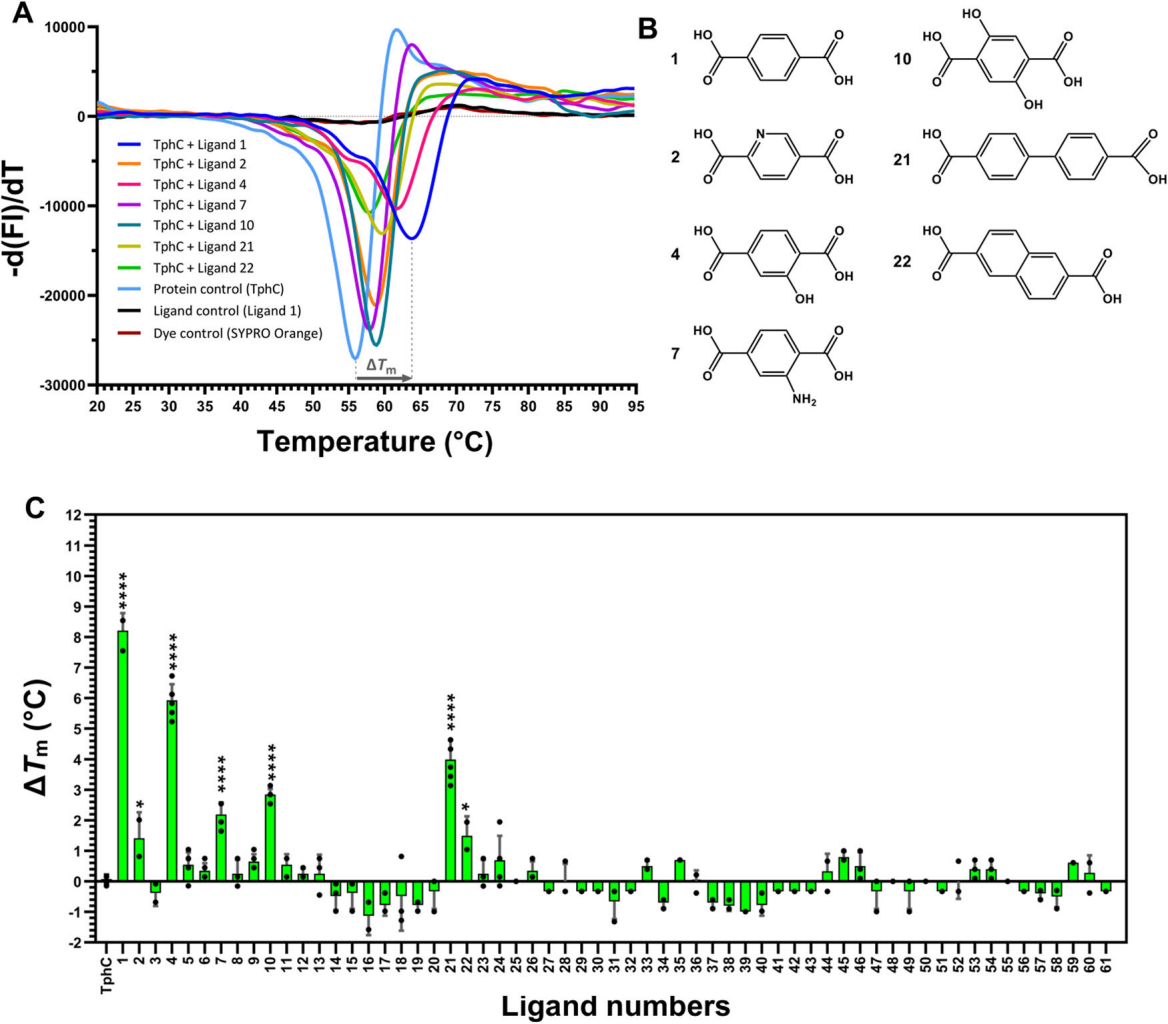
TphC unfolding profile and melting temperature determination. **A** The presence of different ligand induced shifts of the TphC melting curve (light blue) to the right (mauve to dark blue melting curves) resulting in a maximum stabilisation of ~8 °C. The first derivative of melting curves enables the determination of melting temperature *T*_m_ (inflection point in the raw fluorescence traces, see Supplementary Fig. 2. FI fluorescence intensity). **B** Chemical structures of the ligands producing a thermal shift terephthalate (**1**), 2,5-pyridinedicarboxylate (**2**), 2-hydroxyterephthalate (**4**), 2-aminoterephthalate (**7**), 2,5-dihydroxyterephthalate (**10**), bipheny-4,4′-dicarboxylate (**21**) and 2,6-naphthalenedicarboxylate (**22**). **C** The degree of protein stabilisation in the presence of various ligands was calculated as Δ*T*_m_. The data shown are the mean of independent experiments (*n* = 3) and the error bars show the standard deviation (SD) of the mean. The statistical difference between ligand groups and the ligand-free TphC control was assessed using one-way ANOVA using Dunnetts multiple comparisons tests (*p* value < 0.05), where asterisks denote statistically significant difference (* *p* < 0.02; **** *p* < 0.0001) compared to the control. Noted *p* values **1** <0.0001, **2** 0.0184, **4** <0.0001, **7** <0.0001, **10** <0.0001, **21** <0.0001, **22** 0.0115. Each well of a 96-well plate contained 50 µl of total reaction buffer (25 mM Tris-HCl (pH 7.5)/200 mM NaCl), 60 µM of TphC, 1200 µM of ligand and 1× SYPRO orange dye, and fluorescence was monitored at each 1 °C rise in temperature from 20 to 95 °C.

In order to determine the ligand recognition profile of TphC, we procured and/or synthesised TPA analogues and their salts (Supplementary Table 1). TphC was probed for ligand binding against the set of 61 compounds by DSF assay (Supplementary Table 2). DSF can be used to rapidly screen compounds that bind to a macromolecular protein through an apparent stabilisation against thermal denaturation, observed as a shift in the midpoint of the melting curve (Fig. 2 and Supplementary Fig. 2). TphC showed the most stabilised binding interaction with terephthalate (**1**) with an apparent Δ*T*_m_ of 8.3 ± 1.1 °C compared to TphC alone (56.1 ± 0.5 °C). Significant TphC-stabilising interactions were also observed with 2-hydroxyterephthalate (**4**), 2-aminoterephthalate (**7**), 2,5-dihydroxyterephthalate (**10**), and bipheny-4,4′-dicarboxylate (**21**) with Δ*T*_m_s of 5.9 ± 0.7 °C, 2.2 ± 0.6 °C, 2.8 ± 0.4 °C and 4 ± 0.8 °C, respectively. The presence of 2,5-pyridinedicarboxylate (**2**) and 2,6-naphthalenedicarboxylate (**22**) have less pronounced, but still significant, stabilising interactions with TphC, displaying Δ*T*_m_s of 1.4 ± 1.1 °C and 1.4 ± 0.7 °C, respectively. The other para-substituted dicarboxylate analogues (**3, 5–6, 8–11**) regioisomers (**12–13**), hetero-aromatics (**14–19**), bicyclic aromatics (**20, 23**), the mono-carboxylate and carboxylate isosteres (**24–44**), unsaturated phenylpropanoates (**45–50**), phenols (**51–52**), aromatic esters (**53–56**) and aliphatic dicarboxylates (**57–61**) had either negligible effect on Δ*T*_m_s or their interactions with TphC were found to be slightly destabilising under the assay conditions. This indicates that for optimal interaction a six-membered para-substituted aromatic dicarboxylate is required,  plus limited additional substitution by hydroxyl groups around the ring and minor heteroaromatic modifications are tolerated, and also extended aromatic systems are partially tolerated.

### Ligand binding assessment with ITC

**Fig. 3:**
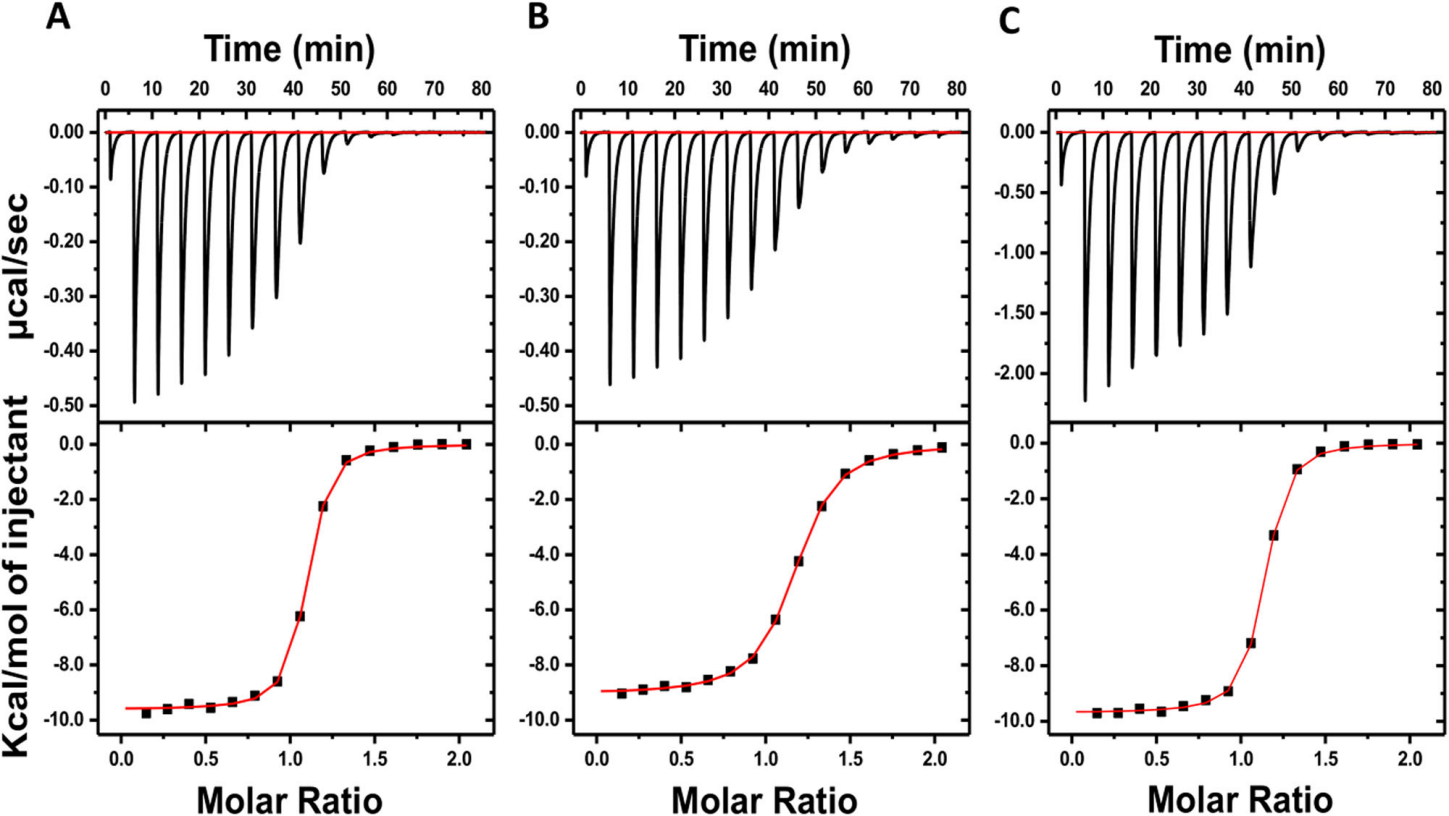
Isothermal titration calorimetry of ligand-hits with TphC. To ascertain the binding interaction of ligand hits and to determine the corresponding thermodynamic parameters for the interactions between the ligands and TphC, isothermal titration calorimetry was employed. **A** Terephthalate **1** against 100 μM TphC. **B** 2-Hydroxyterephthalate **4** against 100 μM TphC. **C** 2,5-Dihydroxyterephthalate **10** against 500 µM TphC. Experiments were performed at 22 °C at a fixed protein to ligand ratio of 1:1:10 in Tris-HCl buffer (25 mM Tris-HCl, pH 7.5/200 mM NaCl), with 2.5 μL ligand injections with 300 s interval between each injection. Corrected heat rates are shown in the top panel and normalised fit to the data in the bottom panel.

The initial DSF assay screening with a variety of aromatic and aliphatic ligands by thermal-shift analysis revealed the ligand-preference of TphC, with the TPA (**1**) and its close structural analogues showing the highest thermal-shift among the ligands tested. To further obtain the thermodynamic parameters for the binding affinity of the ligand hits, ITC experiments were performed (Fig. 3 and Supplementary Fig. 3). The determined *K*_D_ values for the ligands reveal receptor affinities in the µM range and a ligand preference similar to the one observed in the initial DSF screen, with terephthalate (**1**) and its derivatives: 2-hydroxyterephthalate (**4**) and 2,5-dihidroxyterephthalate (**10**) showing the highest affinities with *K*_D_ ranging from 0.36 to 1.9 µM (Table 1). Over two to three orders of magnitude weaker affinities were observed for 2-aminoterephthalate (**7**) and 2,5-pyridinedicarboxylate (**2**) than for terephthalate (**1**), and a considerably lower affinity for 2,6-naphthalenedicarboxylate (**22**). The TphC-ligand interactions for these six ligands were enthalpically favoured and most ligands (Table 1), with the exception of **22**, reported a small entropic penalty (−*T*Δ*S*) upon interaction, indicative of conformational change or solvation effect^54,55^. The reduced affinity observed for **2**, **7** and **22** emphasises the structural constraint for a six-member aromatic ring with a pair of para-oriented carboxylate groups for optimal coordination in the binding site (Supplementary Fig. 4). This is further evidenced with the attempts to titrate TphC with a bulkier biphenyl-4,4′-dicarboxylate (**21**), not displaying any heat of binding, suggesting that **21** has a much lower affinity for ITC assessment or displays an entropically driven interaction. To rule out nonspecific interactions, the specific binding of **21** to TphC was indirectly ascertained through a competitive titration of **1** with TphC saturated with a 10-fold excess concentration of **21** (Supplementary Fig. 5), where the presence of **21** completely abolishes TphC–terephthalate interaction.

**Table 1:** Thermodynamic parameters for binding interactions of ligand-hits with TphC.

Ligand	N sites^a^	*K*_D_ (µM)^b^	Δ*G* (cal/mol)	Δ*H* (cal/mol)	*T*Δ*S* (cal/mol)
Terephthalate (**1**)	1.04	0.364	−8672.28	−9602 ± 38.92	−885.45
2-Hydroxyterephthalate (**4**)	1.13	1.246	−7976.70	−9054 ± 31.21	−1077.30
2,5-Dihydroxyterephthalate (**10**)	1.08	1.931	−7715.59	−9699 ± 27.09	−1983.41
2-Aminoterephthalate (**7**)	1.14	119.9	−5294.95	−5493 ± 43.00	−198.05
2,5-Pyridinedicarboxylate (**2**)	1.01	90.9	−5458.29	−5963 ± 40.74	−504.71
2,6-Naphthalenedicarboxylate (**22**)	1.14	869.6	−4133.46	−3614 ± 134.3	519.46

^a^N represents the stoichiometric ratio of protein:ligand interaction.

^b^A higher affinity for terephthalate disodium and its 2-hydroxy- and 2,5-dihydroxy derivatives was observed.

### Structure of open apo-TphC structure

**Fig. 4:**
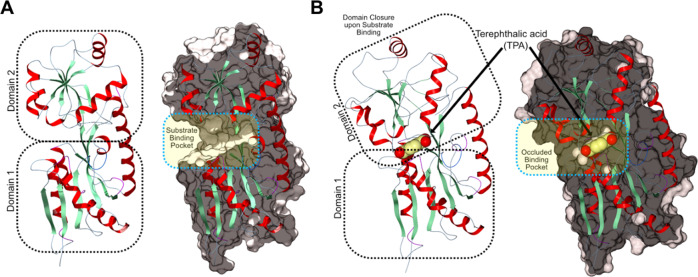
Open and closed structure of the tripartite tricarboxylate transporter—solute binding protein TphC. **A** Crystal structure of TphC is shown in ribbon representation (α-helices red, β-strands green), the structure is comprised of two domains with a large solvent accessible cleft located at the domain interface. A surface representation of the protein has been cut away to highlight the substrate-binding pocket. **B** Upon TPA binding (CPK sphere representation, all atom colours) the two domains reorientate to enclose the bound TPA. The cut away surface representation highlights the occluded pocket in which the TPA is bound.

In order to understand the open structure of TphC, we sought to crystalise TphC alone. Purified TphC was readily crystallised, apo-TphC crystals diffracted to a resolution of 1.97 Å and structure determination via molecular replacement was performed in Phaser using a model derived from TctC from *Polaromonas* sp (PDB 4X9T). The overall structure of TphC maintains the general architecture of two α/β globular domains linked by a pair of β-strands as previously reported in TTT-SBPs TctC (4X9T) and Bug27 (2QPQ) (Fig. 4A and Supplementary Fig. 6). Data collection and refinement statistics for the apo-TphC structure are summarised (Supplementary Table 3). Residues (P28-D37, PFSAGGTAD) map to the motif [P∗-F-X-A-G∗-G∗-X-X-D∗] which is highly conserved in TTT-SBPs (conserved residues underlined)^46^. In the open apo conformation TphC has a solvent accessible surface area of ~13048 Å^2^ with a large solvent accessible cavity at the predicted ligand binding site^51^.

### Structure of closed-TPA-bound TphC

In order to better understand recognition of the TPA ligand and any conformational change associated with ligand binding, we sought to crystalise TphC in complex with TPA. Purified TphC was readily co-crystallised in the presence of 2 mM TPA and crystals diffracted to 2.4 Å resolution. The structure was solved using molecular replacement with a search model derived from AdpC from *Rhodopseudomonas palustris* (PDB 5OEI). The overall structure of the TphC in complex with TPA is shown in Fig. 4B. Supplementary Table 4 summarises the data collection and refinement statistics for the closed TphC–TPA complex. The general architecture of the previously reported TTT-SBPs is maintained in the TphC–TPA structure. Sequence alignment, secondary structure features and conserved motifs for TphC, AdpC and the other TTT-dependent SBPs in the closed conformation are shown (Supplementary Fig. 7 and Supplementary Table 5).

**Fig. 5:**
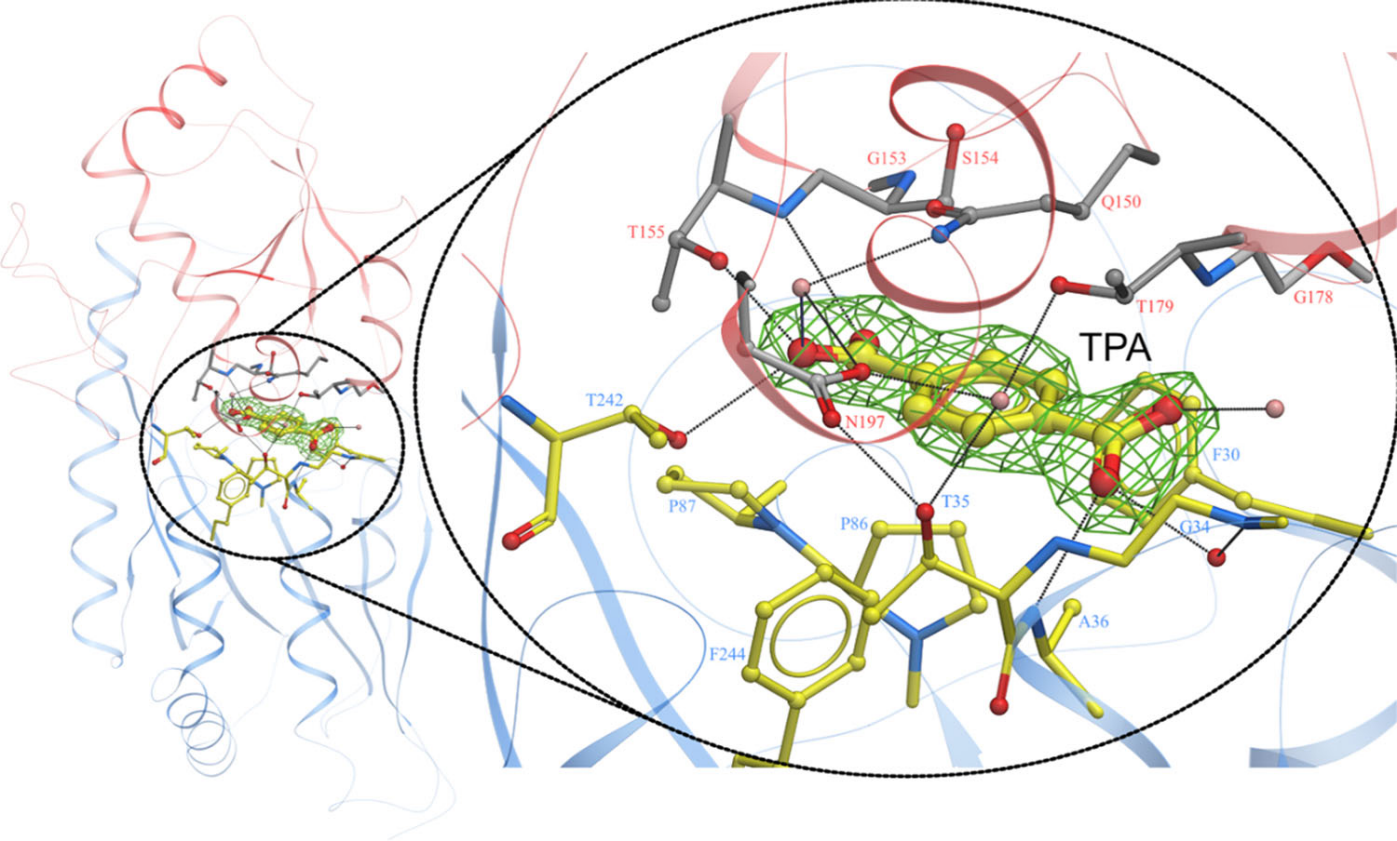
TphC in complex with terephthalate. TphC crystal structure shown in ribbon representation and coloured by domain (domain 1 blue, domain 2 red). A close up of the bound TPA is shown in ball and stick representation (all atom colours) along with its associated 2.4 Å Fo−Fc omit electron density contoured at 2*σ*. The binding pocket interactions are highlighted with residues arising from domain 1 shown in all atom colours (yellow carbon atoms) and domain 2 (grey carbon atoms).

**Fig. 6:**
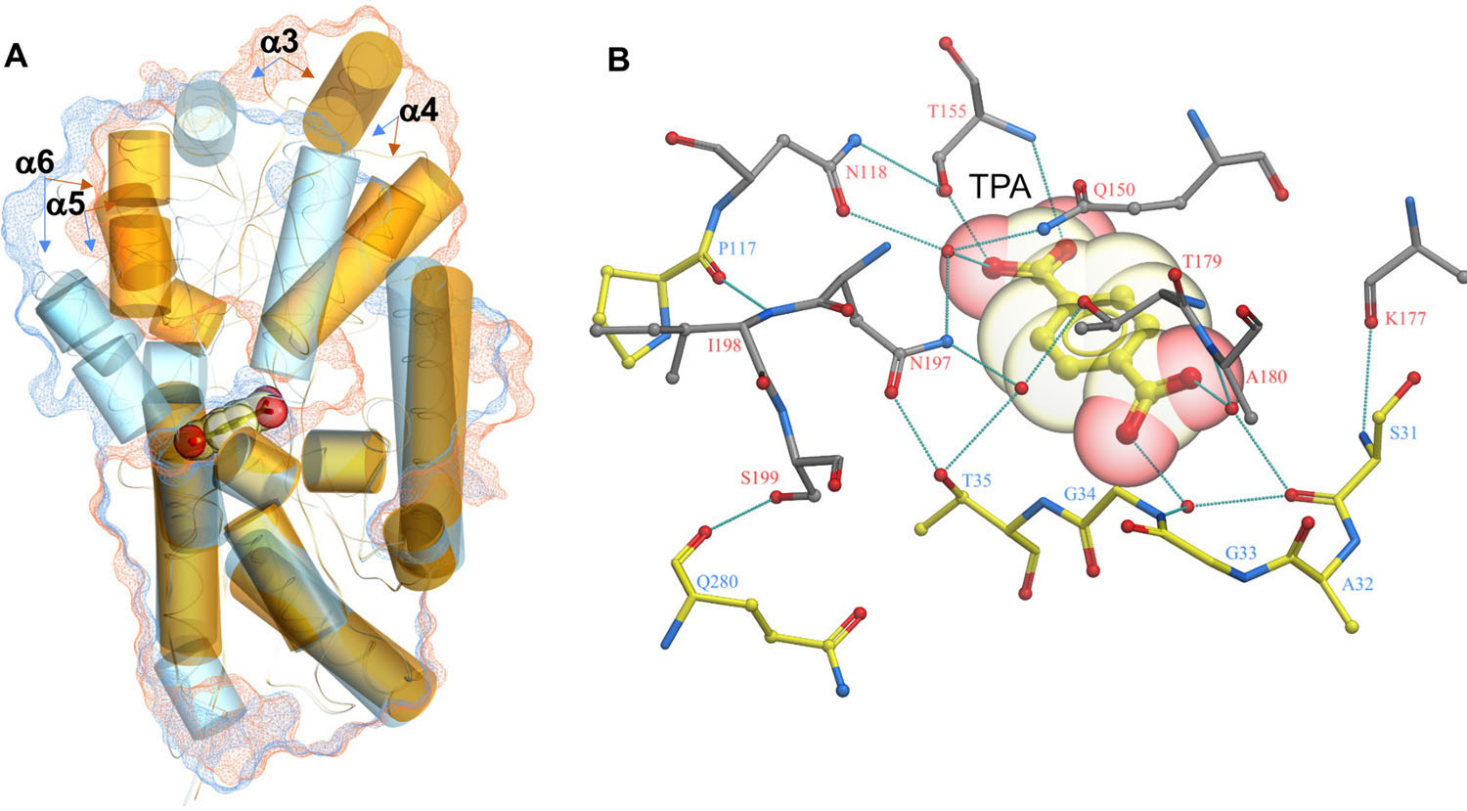
Structure of TphC showing ligand-induced changes. **A** TphC crystal structure in both an apo (open—orange) and TPA-bound (closed—blue) conformation have been superimposed (SSM superposition of domain 1) in ICM-Pro. The protein surfaces for each of the two states are shown as meshes and coloured according to their respective structure (open—orange, closed—blue, the surface representations correspond to thin slices through the surface centred on the TPA-binding site). Helical elements within the structure have been highlighted as large cylinders to clearly show the reorientation between domains 1 and 2 which occur upon TPA binding. TPA is shown in both ball and stick and semitransparent CPK spheres (all atom colours). **B** A ball and stick representation of the TPA-binding site along with highlighted hydrogen bonds that form both between the protein and the TPA and also between the protein domains upon TPA binding. Residues arising from domain 1 are shown with yellow carbon atoms while those from domain 2 are shown with grey carbon atoms.

Superposition of the open and closed TphC structures highlights an ~36° closure between the two domains upon TPA binding. DynDom analysis^56^ identifies residues 115–117 and 241–242 as the hinge region. Upon closure the solvent accessible surface area of TphC decreases from 13,048 to 12,495 Å^2^ encapsulating a closed cavity of 280 Å^3^ in which the TPA is bound. Hydrogen bonds between both the proximal and distal carboxylate groups of the TPA stabilise the closed conformation of the protein along with a network of direct and water-mediated hydrogen bonds highlighted in Fig. 5. The bound TPA is recognised through a number of direct and water-mediated hydrogen bond interactions. The TPA carboxylate group buried deepest within the pocket is recognised and stabilised through direct (T155, T242) and water-mediated hydrogen bonds (T155, Q150, N197). The second carboxyl group of the TPA hydrogen bonds to the backbone amino groups of A36 and with additional water-mediated interactions with the backbones of G34, S31 and T179. The interactions at this second carboxylate group span between the two TphC domains stabilising the closed conformation (Fig. 5). The binding pocket is bordered on one side by a conserved aromatic residue (F30); the positioning of this sidechain is facilitated by the proximity of a conserved glycine (G178). Unlike other TTT-SBPs characterised to date, the ligand site of TphC contains bulky amino acids P86 and Q150, the sidechains of which create a very narrow pocket 6.4 Å across, which would appear to confer the observed selectivity toward planar aromatic ligands. In addition, the Q150 sidechain is closely bounded by S154 (in addition to S156 and F194 not shown), all of which together would prevent alternative rotamers being sampled to increase the diversity of ligands accommodated. Overlaying the open and closed TphC structures indicates the movement of the upper domain (α4–α6) (Fig. 6A). Upon closure a pincer is formed between loops on the lower (β1–α1) and upper domains (β6–α5) forming additional H-bonds directly between S31 and K177, T35 and N197, and water-mediated contacts and between T35 and T179 (Fig. 6B).

### Docking and TPA depletion

In order to visualise their potential binding site interaction, the seven ligands were docked into the binding site of the closed TphC, terephthalate **1**, 2,5-pyridinedicarboxylate **2**, 2-hydroxyterephthalate **4**, 2-aminoyterephthalate **7**, 2,5-dihydroxyterephthalate **10**, biphenyl-4,4′-dicarboxylate **21** and 2,6-naphthalenedicarboxylicacid **22** (Supplementary Fig. 8 and Supplementary Table 6). To investigate the transport of the selected analogues in vivo, we performed substrate depletion assays with resting cells of *P. putida* KT2440 ∆*pcaGH* harbouring the plasmid pJCBAtG^45^. As previously shown^45^, resting cells were able to transport and thus deplete TPA by ~20% after 60 min (Supplementary Fig. 9). However, due to the lack of their enzymatic conversion, neither depletion nor transport of the selected analogues (**2**, **4**, **7**, **10**, **21** and **22**) could be determined. Further experimentation would help investigate the transport of these ligands by TphC in vivo.

### Mutational analysis of amino acids involved in ligand binding

In order to confirm the involvement of the amino acids in TPA and analogue recognition, and ligand-induced conformation change a series of mutant proteins were created and screened with DSF. This included mutation of amino acids involved in recognition of TPA via direct H-bond interactions (Fig. 5), T155A, T242A, T155A/T242A, those involved in creating a narrow binding pocket Q150S, P86T/Q150S, along with an amino acid within the hinge region V116P. Mutation of the amino acids involved in direct ligand recognition had little or no impact (<3 °C) on the intrinsic stability of the TphC as measured by *T*_m_; however, the P86T/Q150S and Q150S displayed a higher *T*_m_ (>3 °C) indicating higher intrinsic thermal stability (Supplementary Fig. 10A). In addition, the hinge mutant V116P displayed a significantly lower *T*_m_ (<10 °C) indicating lower intrinsic thermal stability. The seven most active ligands (**1**, **2**, **4**, **7**, **10**, **21** and **22**) were then screened against the protein mutants (Supplementary Fig. 10B). The loss of H-bonding potential for T155A is clearly observed for all substrates where no significant ligand-induced stabilisation was observed. Unexpectedly the T242A mutant still bound to TPA and ligand **4** indicating the less stringent requirement of T242 in substrate recognition. The double-mutant T155A/T242A also displayed no ligand-induced stabilisation in the presence of TPA or the analogues consistent with the single mutant T155A. A small, but still significant shift was observed in the presence of TPA towards Q150S, although the other binding pocket mutant, P86T/Q150S, was no longer able to bind TPA. Finally, the hinge mutant V116P displayed ligand-induced stabilisation in the presence of TPA and five of the analogues (**4**, **7**, **10**, **21** and **22**); for TPA this stabilisation (Δ*T*_m_) was greater than that observed with the wild-type TphC. This greater shift can be explained by the lower intrinsic thermal instability of the V116P (Supplementary Fig. 10A).

### Phylogenetic analysis of TphC homologues and genomic context analysis

**Fig. 7:**
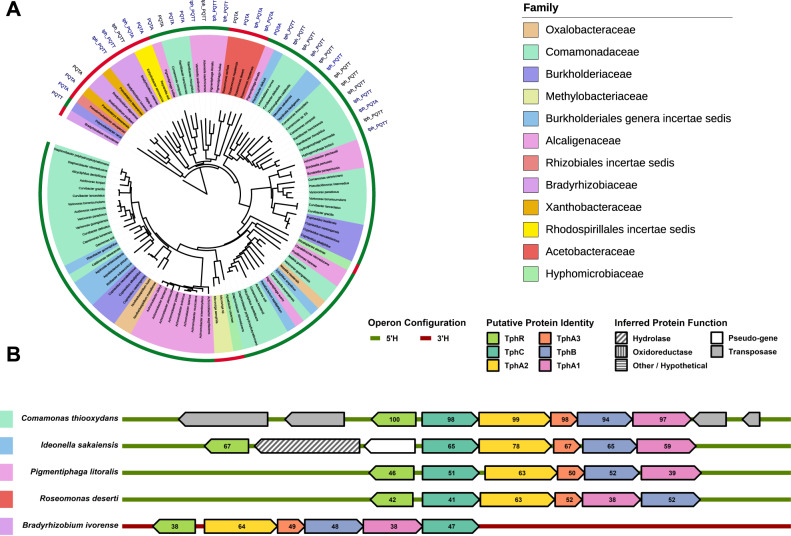
Anotated phylogenetic tree of TphC and configuration of putative *tph*-like operons. **A** Phylogenetic tree of TphC constructed by retrieving, aligning and displaying sequences (using BLAST-P, Clustal Omega and iTOL respectively). Species names are coloured by taxonomic family (*n* = 12) and taxonomic class is shown by a coloured outer ring (α-proteobacteria (red) and β-proteobacteria (green). Genomic loci proposed to be *tph*-operon homologues are anotated (tph). Additionally, the amino acids (and their homologue equivalent) directly involved in ligand binding within the TphC-TPA are labelled, where P86, Q150, T155, and T242 (or A242) are labelled as PQTT (or PQTA). **B** Schematic diagram of the operon configuration for a selection of putative *tph*-like operons. Taxonomic family is shown by coloured boxes to the left of the operon. The values inside the genes indicate the percentage similarity of the encoded protein to the corresponding protein in *Comamonas* sp. strain E6. These operons show a range of alternative features, such as the presence of genes encoding transposases (*Comamonas thiooxidans*), a hydrolase (*Ideonella sakaiensis*), and alternative operon architectures (*Roseomonas deserti* and *Bradyrhizobium ivorense*).

The gene encoding TphC is located in the *tph* operon in *Comamonas* sp. strain E6, which is composed of the transcriptional regulator (*tphR*), solute-binding protein (*tphC*), TPA dioxygenase subunit A2 (*tphA*_*2*_), TPA dioxygenase subunit A3 (*tphA*_*3*_), TPA dioxygenase subunit B (*tphB*), TPA dioxygenase subunit A1 (*tphA*_*1*_) (Fig. 1A). Phylogenetic analysis of TphC from *Comamonas* sp. strain E6 identified SBP homologues (*n* = 100), with sequence identity ranging from 39 to 98% relative to the query (Fig. 7). Taxonomic analysis reveals that the entries originate from 80 different species, 46 genera, 12 families, and majority of the entries (81) originate from species belonging to the Burkholderiales order (β-proteobacteria), 13 to the Rhizobiales order (α-proteobacteria), and 6 to the Rhodospirillales order (α-proteobacteria) (Supplementary Data 1). Due to the environmental and biotechnological importance of TphC and the associated genes in the uptake and catabolism of TPA, we analysed the gene content, operon architecture and encoded protein homology of the operons flanking the *tphC* homologues (Fig. 7 and Supplementary Fig. 11). The *tph* operon content was conserved in a subset of these selected entries (22/100), including from *Ideonella sakaiensis*, representing species from five different families within the α- and β-proteobacteria classes (Supplementary Data 1). TphC homologues encoded by the *tph*-like operons contained highly conserved amino acids at the positions where the sidechains make direct contact with TPA (T155, T242) or create the planar binding pocked (P86, Q150) (Fig. 7). A small subset of operons encoding TphC-like proteins contain A242 rather than T242, consistent with the observed non-essential nature of T242 in TPA recognition (Supplementary Fig. 10). Interestingly the same conserved binding site amino acids are also present in TphC homologues not associated with *tph*-like operons (Fig. 7A), indicating alternative genomic loci as sources of potential novel TPA catabolic genes.

Homology alignment of the TphC sequences associated with the *tph*-like operons indicated the highly conserved amino acids within the ligand binding site (Supplementary Fig. 12). Sequence identity, represented by calculating the percentage identity for each protein type across the *tph*-like operons, was generally modest across all proteins (62%), with TphR the least conserved (55%) and TphA2 the most conserved (77%) (Supplementary Fig. 13). Within these operons the predominate operon architecture was the same as that found in *Comamonas* sp. strain E6. Here *tphC* is located at the 5′ end of the operon and *tphR* divergently located (head-to-head), with respect to the corresponding promoter (5′H). This 5′H operon structure is observed in both α- and β-proteobacteria classes (e.g. *Comamonas thiooxidans* and *Roseomonas deserti*) (Fig. 7B and Supplementary Fig. 11). Within an alternative operon structure, *tphC* is located at the 3′ end of the operon, this operon architecture was observed exclusively in α-proteobacteria (e.g. *Bradyrhizobium ivorense*) (Supplementary Data 1). Within these genomic loci the presence of transposable elements flanking the *tph* operon within *Comomonas thioxydans* and other operons is indicative of horizontal gene transfer of the *tph* operon reflecting the accessory nature of these genomic loci (Fig. 7B) (Supplementary Fig. 11). Additionally a number of operons contained gene insertions within the *tph* operon encoding for a hydrolase (MHETase), oxidoreductase, and a transporter (e.g. *Comamonas composti*, *Ideonella sakaiensis*, and *Piscinibacter defluvii*), presumably involved in TPA recognition and catabolism.

## Discussion

In this study, we characterised the TphC SBP that is a key component in the cellular uptake of TPA, the breakdown product of PET plastic^45^. TphC belongs to the TTT family in *Commamonas* sp. strain E6 and through biochemical and structural analysis, the ligand-binding properties and 3D structure were determined in both open and ligand-bound closed forms. TTT-dependent SBPs, such as TphC belong to the Cluster E-II according to the classification of Scheepers et al.^57^. As with the tripartite ATP-independent periplasmic (TRAP) class of SBP-dependent transporters, they are symporter proteins driven by an electrochemical ion-gradient, rather than ATP as per the SBP-dependent class of ABC transporters^46^.

Screening with DSF showed TphC binds to para-substituted aromatic dicarboxylates. Substitution of the aromatic ring with hydroxyl and amino groups, and the presence of hetero-atoms within the ring, is tolerated, but with a significantly lowered ligand-induced stabilisation. This seems to indicate that the binding pocket of TphC is structurally quite constrained. Furthermore, the efficient binding of the terephthalate **1** ligand but not the C_6_ aliphatic or unsaturated aliphatic ligands, adipate **59** and *trans,trans*‐muconate **57**, revealed the need for an aromatic structure for the binding ligand. The TTT-SBPs characterised so far are specific for aliphatic ligands^47,49,50^ with the sole exception of the *B. pertussis* Bug27 protein which is reported to bind an aromatic mono-carboxylated nicotinate^51^. Our model proposes that TphC has a narrower more selective binding site, whereas AdpC is more relaxed, which is consistent with biochemical data in the literature that shows that AdpC binds to C_6_–C_8_ aliphatic dicarboxylates plus *trans, trans* muconate^47^. The ITC determined TphC affinities for the ligand-hits were in µM range with terephthalate **1** and its derivatives: 2-hydroxyterephthalate **4** and 2,5-dihidroxyterephthalate **10** showing the greatest affinities (0.36−1.9 µM). The sub-micromolar TphC affinity observed for **1** (0.36 µM) is comparable to other high-affinity SBPs reported for Bug27-Nicotinate/Nicotinamide (0.36 µM)^51^, AdpC-Adipate (0.55 µM)^47^ and MatC-Malate (~0.02 µM)^48^.

In comparison with other open/apo structures, even though TphC has low similarity and identity to other apo SBP structures TctC (26%) and Bug27 (29%), the two domains of TphC have RMSD of 0.837 and 0.446 for the corresponding domains within TctC (PDB 4X9T), and 0.47 and 0.891 within the Bug27 (PDB 2QPQ) domains (Supplementary Table 5). All three SBPs have the same architecture; however, there is significant differences between the orientation of domains between TphC and TctC, specifically in the upper domains around α5-6 and β5-8, and the ligand-binding loop between β6 and α5 (Supplementary Fig. 6). TphC shows a 36.3° closure angle, whereas previously resolved apo- and ligand-bound structures of Bug27 reported a closure angle of 24.7°^51^ and other periplasmic binding proteins show closure angles ranging from 15° to 60°^58^. Similar to MatC and AdpC, the absence of positively charged residues (to counteract the negative charges of the ligand carboxylate groups) in the ligand-binding pocket of TphC is observed. The TphC–TPA ligand-binding site appears similar to the aliphatic-SBP complex (oxoadipate-AdpC), with comparable distances between the corresponding residues responsible for H-bonding with the dicarboxylate groups. Within SBPs that recognise the smaller aspartate and malate ligands (BugD and MatC), the C_4_ ligands are positioned 90° rotated along the dicarboxylate axis relative to TPA (and the other C_6_ ligands), with the carboxylate H-bonding to a neighbouring serine residue (S151). While this serine is conserved in TphC (S156), a bulky glutamine residue in TphC (Q150) occludes the alternative H-bonding site and thus provides the rationale for why the smaller dicarboxylate ligands are not recognised by TphC. The bulky residues P86 and Q150 present within TphC create a narrow pocket, 6.4Å across, which appears to exclude aliphatic C_6_ ligands.

Along with the majority of TTT-SBPs reported to date^46^, TphC and its homologues identified here are not located in the same operon as the TTT-dependent membrane transporter subunit genes (*tpiA* and *tpiB*), but are located within the *tph* operon along with the genes encoding transcriptional regulator (TphR) and the TPA dioxygenase subunits (TphA_1–3_B) (Fig. 7). Due to the genomic co-localisation of the *tphC* and the catabolic pathway for TPA (*tph*), we sought to use TphC as genomic fish-bait to locate other homlogous gene clusters of biotechnological interest. This approach has previously been employed to identify arylmalonate decarboxylase^59^.

PET was first synthesised in the 1940s from TPA and EG, and TPA in turn is prepared by the chemical oxidation of *p*-xylene^60^. In addition, TPA is used in the chemical synthesis of small-molecule plasticiser additives^61^, which may be more accessible sources of TPA within the environment rather than from highly crystalline PET^62,63^. As far as we know, TPA is not found naturally in the environment, so the question exists as to from where did the TPA recognition potential originate. At the outset of this study we had expected to identify closely related chemical compounds of natural origin that bound to TphC. However, all TphC-binding ligands identified in our screen appear to be of xenobiotic origin—why binding to non-xenobiotic compounds was not observed is unclear. One possibility is that TphC evolved from an ancestral or pre-industrial SBP that displayed side-activity or promiscuity, and TphC has since lost affinity to its original ligand, as has been proposed for the evolution of enzymes^64^. Considering the evolutionary time span of ~80 years since the first production of TPA, and the low conservation observed between the SBP’s associated with the putative *tph* operons (Fig. 7), it would seem likely that TphC homologues might have evolved from a pool of multiple ancestral SBPs rather than from a common ancestor. This notion is also consistent with the low degree of sequence conservation and global distribution of xenobiotic degradation pathways, e.g., those involved in plastic^21,65^ and organohalide^66^ degradation.

In this study, we provide evidence for the structural basis of TPA recognition by TphC through DSF, ITC and X-ray crystallography. Together with TPA, we report the binding potential of TphC towards some other TPA derivatives and analogues which have varied applications. For instance, 2,5-dihydroxyterephthalic acid is a useful monomer for the synthesis of high strength fibres^67^, 2-aminoterephthalic acid and 2,5-pyridinedicarboxylic acid have application in the synthesis of lanthanide coordination polymers^68,69^ and 2,6 naphthalene dicarboxylic acid is used as a monomer for the production of polyethylene naphthalate (PEN) esters which are considered superior to PET for certain applications^70^. In summary this biochemical, structural and phylogenetic analysis provides useful insights into the ligand-recognition potential of TphC and opens up a new array of opportunities to engineer heterologous hosts for the uptake and assimilation of the breakdown products from PET and other polymers.

## Methods

### Chemicals, genes and reagents

All reagents were procured at the highest purity available from Sigma Aldrich Ltd. (Dorset, UK) unless otherwise specified. Genes were synthesised by IDT DNA technologies, and molecular biology reagents were purchased from NEB unless otherwise stated.

### Cloning, Expression and Purification of TphC

The *tphC* gene from *Comamonas* sp. E6 (BAE47076.1), excluding the first 26 amino acids of the TphC protein (WP_019043844.1) comprising of the N-terminal signal sequence, was chemically synthesised with the codons optimised for expression in *E. coli*. The cloning of *tphC* was carried out using Gibson assembly of linearised pET44a(+) plasmid with the synthetic truncated DNA sequence of *tphC*. The resultant plasmid was transformed into *E. coli* DH5α and subsequently into *E. coli* expression host BL21(DE3) for overexpression. The cells bearing the plasmid pET44a-his-TphC were initially grown at 37 °C with shaking (200 r.p.m.) in terrific broth medium supplemented with 100 µg/mL ampicillin and 0.2% (w/v) glucose. The overnight seed culture was then equilibrated to 25 °C and then 200 mL was used to inoculate 22 L auto-induction medium, pre-equilibrated to 25 °C, consisting of (w/v): 2% tryptone, 0.5% yeast extract and 0.5% NaCl and supplemented with 0.4% (v/v) glycerol, 0.002% (w/v) glucose and 0.01% lactose and 100 μg/mL of ampicillin. The culture was grown at 25 °C for 48 h. The culture was centrifuged at 5000*g* at 4 °C for 1 h and the cell pellet was re-suspended (100 g wet cells/200 mL buffer) in the binding buffer (25 mM Tris-HCl, pH 7.5/500 mM NaCl/10 mM imidazole). The cells were lysed using a pre-chilled CF1 cell disrupter (Constant Systems Ltd, UK) in the presence of EDTA-free protease-inhibitor cocktail (Roche) and endonuclease (Benzonase) and the lysate was centrifuged at 125,000*g* for 1 h. The resulting supernatant was loaded onto a 5 mL Ni-NTA agarose column, pre-equilibrated with 10-column volumes of binding buffer. The column was then washed with 5-column volumes of washing buffer (25 mM Tris-HCl, pH 7.5/500 mM NaCl/20 mM imidazole) and the bound TphC was step-eluted with 3 × 2-column volumes of elution buffers containing increasing concentrations of imidazole (25 mM Tris-HCl, pH 7.5/500 mM NaCl/40-150 mM imidazole). To remove imidazole and to concentrate the protein, the eluted TphC fractions were pooled and buffer exchanged into a Tris buffer (25 mM Tris-HCl, pH 7.5/200 mM NaCl) using PD-10 columns. The buffer-exchanged TphC protein was concentrated with a centrifugal filter concentrator (Amicon, 10 kDa MWCO, Merk Millipore) and subjected to gel-filtration chromatography on a high-performance gel-filtration column (AKTA, Superdex-200 26/600 GL, GE Healthcare) previously equilibrated with the Tris buffer. The peak fractions from gel-filtration column were collected and assessed for purity on SDS-PAGE. The protein concentrations were determined from the calculated extinction coefficient of TphC at 280 nm. The mid-peak fractions were pooled and concentrated, as described above, to 5–10 mg/mL and the concentrated protein was stored in 100 µL aliquots at −80 °C until later use.

### Ligand synthesis

Most of the TPA analogues are insoluble in aqueous solution, but are soluble in DMSO which makes it difficult to assess them using ITC. As a result, a majority of the ligands were sourced as the sodium salt or the sodium salt was synthesised from the free acid when the salt was not commercially available. Nineteen sodium salts were prepared as described: organic acid (3 mmol) was dissolved in NaOH solution (3 mL, 2 M), this solution was slowly added to acetone (40 mL), precipitating the sodium salt. The solid was isolated by filtration, washing with acetone (3 × 5 mL), and remaining solvent removed in vacuo, affording dry solid product. Elemental Analysis was performed and the purity calculated (Supplementary Table 1).

### DSF assay

TphC protein–ligand interactions were investigated using a DSF assay^47^. Preliminary assays were conducted to determine a minimum TphC concentration required in the assay to achieve a fluorescence signal strength above the noise in the assay. The assays were conducted at a fixed protein to ligand ratio of 1:10 between the protein concentration range of 5–1000 µM in a 96-well plate in a total reaction volume of 50 µL buffer (25 mM Tris-HCl, pH 7.5/200 mM NaCl) containing 1× concentration (5 µL, 10×) of SYPRO orange dye. The assays were run on a QuantStudio 3 Real-Time PCR System (ThermoFischer Scientific, UK) with melt curve experiments equilibrating to 20 °C before ramping to 95 °C while recording fluorescence at every 1 °C rise in the assay temperature. Under the assay set-up a TphC concentration of 60 µM was determined to be the optimal concentration for the assay. The subsequent ligand screens were set up as follows: ligand (5 µL, 1200 µM), protein (10 µL, 60 µM), SYPRO orange dye (5 µL, 10×) and Tris-HCl buffer to a total volume of 50 µL. Where the ligand was dissolved in DMSO the same concentration (v/v) of DMSO was added to the parallel controls. Data were processed in excel and analysis in Prism 8 (GraphPad).

### Isothermal titration calorimetry

All ITC measurements were carried out using a MicroCal Auto-iTC200 (Malvern Panalytical Ltd, UK) set in a passive mode with a reference power of 4 µcal/s. For isothermal calorimetric titration, TphC concentration was adjusted to either 100 or 500 µM and the ligand solution to 1 or 5 mM in the same stock of Tris buffer as was used during the size exclusion chromatographic purification. The buffer (800 µL/well), protein (400 µL/well) and ligand (200 µL/well) solutions were dispensed into a 96-well sample tray and allowed to temperature-equilibrate for 30 min before being auto-dispensed to the reaction cell and the titrating syringe. The active reaction cell contained 300 µL of 100 µM protein and the syringe contained 120 µL of 1 mM ligand. The protein–ligand titrations were carried out at 22 °C with an initial pilot injection of reduced volume (0.5 µL, 1 s) followed by 15 successive injections (2.5 µL, 5 s) spaced 300 s apart. Reference buffer to buffer titrations were run in series and were subtracted from the test titrations. The resulting data were processed and an independent binding model was fit in ITC data analysis software (OriginLab Corporation).

### Ligand depletion assays

The plasmid pJCBAtG^45^ developed previously was transformed into *Pseudomonas putida* KT2440 ∆*pcaGH*, which is unable to metabolise protocatechuate. The pJCBAtG plasmid carries *tphC/tpiBA* and the full *tphAB*_*II*_ catabolic operon. Resting cell conversion assays were performed as previously described^45^. Briefly, *P. putida* ∆*pcaGH* cells harbouring pJCBAtG were grown in terrific broth medium supplemented with 25 µg/mL tetracycline for 10 h after induction with *m*-toluate, harvested by centrifugation, washed with 50 mM Tris-HCl buffer (pH 7.5) and re-suspended to an OD_600_ of 40 in the same buffer. Depletion of ligands was investigated in 1 mL assays containing 1 mM of the corresponding substrate and cells to a final OD_600_ of 30 in 50 mM Tris-HCl buffer. The assays were incubated at 30 °C for 60 min. Supernatant samples were collected at time 0 and after 60 min, filtrated and ligand concentration analysed by high-performance liquid chromatography (HPLC; Agilent 1100 HPLC system, Agilent Technologies, UK). Separation was achieved using an Agilent Poroshell 120 EC-C18 column (4 µm, 100 mm, Agilent Technologies, UK) with a gradient 5–95% acetonitrile containing 0.1% formic acid as the mobile phase. All ligands were detected at 254 nm.

### Protein crystallisation, data collection and structure determination

The crystallisation of TphC in the absence of cognate ligand was achieved by mixing 200 nL of 10 mg/mL of the protein in sitting drop vapour diffusion experiments with an equal volume of a range of different commercial screens (Molecular Dimensions Ltd, Newmarket, UK) at 20 °C. The crystals grew with reservoirs of condition B5 from the Morpheus HT96 screen (0.09 M Halogens 0.1 M Buffer system 2 pH 7.5 30% v/v Precipitant mix 1). Prior to data collection single crystals were flash frozen in liquid nitrogen. All data were collected at the Diamond Light Source (Harwell, UK). Data processing was performed with Dials, the apo-TphC structure was solved by molecular replacement in Phaser using a search structure derived from the TctC structure (4X9T). Individual domains were isolated from 4X9T and used together in Phaser to achieve the successful molecular replacement. Structure deposited to PDB 7NDR. The co-crystallisation of TphC with the ligand disodium terephthalate was achieved by adding 2 mM ligand to 10 mg/mL of the protein prior to screening as described above for the apo crystal form. The co-crystals grew with reservoirs of condition B4 from the SG1 HT96 screen (0.2 M ammonium sulfate 0.1 M MES 6.5 30% w/v PEG 5000 MME). Crystals were flash frozen in liquid nitrogen in the presence of mother-liquor plus glycerol (20% v/v; added as cryoprotectant). Data processing was performed with Dials and the TphC-TPA structure was solved by molecular replacement using a search model derived from the AdpC structure (5OEI). Structure deposited to PDB 7NDS. The structure were visualised and annotated using MolSoftPro.

### TphC mutagenesis

The genes encoding mutants Q150S and T242A were codon optimised for expression in *E. coli*, chemically synthesised (gBlocks, IDT Integrated DNA Technologies) and assembled using NEBuilder HiFi DNA assembly master mix (New England Biolabs) into a pET44a(+) backbone. The additional point mutations were created by site-directed mutagenesis (Q5, New England Biolabs) using the corresponding plasmid template (WT, Q150S, or T242A), with the appropriate primers (Supplementary Table 7), and the resulting products were treated with a KLD enzyme mix (NEB) before being transformed into *E. coli* NEB 5-alpha competent cells. The mutations were confirmed by sequencing. The plasmids were transformed into *E. coli* BL21(DE3) and proteins produced as above.

### Molecular docking

Molecular docking was performed on the closed form of TphC (PDB: 7NDS) using Autodock Vina 1.1.2 (ref. ^71^). For the ligands SMILES strings were converted into the PDBQT format using Open Babel 2.4.1 (ref. ^72^) and for the protein a PDBQT file was prepared using AutoDockTools 1.5.6 (ref. ^72^). Docking was performed using a cubic search volume with 30 Å sides centred on the geometric centre of the protein with an exhaustiveness of 25. The average docking for all ligands in the library is −6.13 kcal/mol with a standard deviation of 1.02, compared to the scores for the ligands shown in Supplementary Table 6. To illustrate the putative binding mode for biphenyl-4,4′-dicarboxylate **21**, which was not successfully docked in the crystal structure, the ligand was first positioned in the active site by alignment to the docked pose of the similarly sized 2,6-naphthalenedicarboxylate **22**, after which the system was energy minimised with 1000 steps of steepest descent followed by 10 steps of conjugate gradient in UCSF Chimera 1.14 (ref. ^73^) with the Amber FF14SB force field^74^ for the protein the AM1-BCC atom and bonding definitions for the ligand, and biphenyl-µ4,4-dicarboxylate was then docked into the resulting protein structure. After energy minimisation, the main change in the protein structure was in the loop formed by residues 30–35, with a resulting RMSD of 0.502 Å relative to the crystal structure for these residues (and 0.095 Å for the whole protein).

### Phylogenetic, taxonomic and genomic context analysis

Searching for TphC-like proteins was performed by Basic Local Alignment Search Tool (BLAST) in National Center for Biotechnology Information (NCBI) server using the BLAST-P method. Multiple alignment was performed with Clustal omega, and visualised using iTOL^75^. Taxonomic information for these sequence files was then retrieved using the NCBI Taxonomy page. If there were multiple entries/species associated with a particular TphC-like protein homologue, then the first NCBI database entry was selected. Genomic context analysis was performed by retrieving the corresponding protein sequences from the genes flanking the *tphC*-like genes (±7000 nts) using NCBI database. The resulting sequence files were parsed using the biopython and pandas packages^76,77^ in python and further processed using the tidyverse package R^78^ (Supplementary Table 7).

Homologous proteins were selected on the basis on NCBI annotation, homology analysis was subsequently performed between the putative homologues and *Comamonas* strain sp. E6 using Clustal omega, and genomic loci containing four or more *tph*-like genes were classified as *tph*-like operons. Genes within these operons that could not be classified as *tph-*like had their function inferred using Blast2GO^79^. The operon configuration was annotated on basis of the location of the genes encoding TphR and TphC relative to the corresponding intergenic/promoter region. Predominately *tphC* is located at the 5′ end of the operon and *tphR* located head-to-head with respect to the corresponding promoter (5′H). These data were plotted using R and the tidyverse, gggenes^80^ and RColorBrewer^81^ packages. The graphs produced were finalised using GNU Image Manipulation Program^82^.

### Reporting summary

Further information on research design is available in the Nature Research Reporting Summary linked to this article.

## Supplementary information

Supplementary Information

Description of Additional Supplementary Files

Supplementary Data 1

Reporting Summary

## Data Availability

The X-ray datasets generated during the current study are available in the Worldwide Protein Data Bank (wwPDB) repository with accession codes 7NDR and 7NDS. The Phylogenetic data generated in this study are provided in the Supplementary Data file 1. Source data are provided with this paper.

## Source data

Source Data
