# Supplementary material for: Structural basis of terephthalate recognition by solute binding protein TphC
Source: Nat Commun. 2021 Oct 29;12:6244. doi: 10.1038/s41467-021-26508-0 (PMC8556258; doi:10.1038/s41467-021-26508-0)
Supplement: Supplementary file 1 — Supplementary Information [file 41467_2021_26508_MOESM1_ESM.pdf]

Structural basis of terephthalate recognition by solute binding protein TphC

Supplementary Information

Trishnamoni Gautom, Dharmendra Dheeman, Colin Levy, Thomas Butterfield, Guadalupe Alvarez Gonzalez, Philip Le Roy, Lewis Caiger, Karl Fisher, Linus Johannissen, Neil Dixon

neil.dixon@manchester.ac.uk

Contents

Supplementary Fig. 1A. Alignment of native and recombinant TphC sequences

Supplementary Fig. 1B. Purity of TphC sample after gel-filtration chromatography using Superdex-200 26/600 GL column

Supplementary Fig. 2. Differential scanning fluorimetry of TphC

Supplementary Fig. 3 Isothermal titration calorimetry of ligand-hits with TphC

Supplementary Fig. 4. Calculated distance between dicarboxylates

Supplementary Fig. 5. Competitive interaction of biphenyl-4,4′-dicarboxylate and terephthalate for TphC

Supplementary Fig. 6. Sequence alignment and overlay of open apo-SBP TTT structures

Supplementary Fig. 7. Sequence alignment and identity matrix scores for the closed holo-SBP TTT structures

Supplementary Fig. 8. Docked structures

Supplementary Fig. 9. Ligand depletion assays

Supplementary Fig. 10. Mutational analysis of amino acids involved in ligand binding

Supplementary Fig. 11. Schematic diagram of operon configuration of all putative *tph*-like operons

Supplementary Fig. 12. TphC homology alignment

Supplementary Fig. 13. Sequence diversity of the putative *tph* operons

Supplementary Table 1. Salts synthesised for terephthalate structural analogues

Supplementary Table 2. Ligands investigated for thermal-shift of TphC using DSF assay

Supplementary Table 3. X-ray data collection and refinement statistics TphC (open)

Supplementary Table 4. X-ray data collection and refinement statistics for TphC-TPA (closed)

Supplementary Table 5. Comparison of closed TphC with the TTT-SBP homolog structures available in the PDB.

Supplementary Table 6. Docking scores for selected ligands**.**

Supplementary Table 7. Primers used for Site Directed Mutagenesis

```
TphC_Comamonas_E6      MRNESIRRREALIGIAAAVAATGSLAQSNQPLKIVVPFSAGGTADVLPRLVAEKIRADYA   60
rTphC_E6               --------MGSSHHHHHHGSGENLYFQSNQPLKIVVPFSAGGTADVLPRLVAEKIRADYA   52
                                  :        :. .   **********************************

TphC_Comamonas_E6      GGVIIENKPGAGGNIGADLVFRAPPDGMTVLASPPGPIAINHNLYQKLSFDPTRWVPVTI   120
rTphC_E6               GGVIIENKPGAGGNIGADLVFRAPPDGMTVLASPPGPIAINHNLYQKLSFDPTRWVPVTI   112
                       ************************************************************

TphC_Comamonas_E6      LATVPNVLVINPKLPVKSLGEFIAYAKANPKKVTVATQGDGSTSHLTAAMFMQLTGTELT   180
rTphC_E6               LATVPNVLVINPKLPVKSLGEFIAYAKANPKKVTVATQGDGSTSHLTAAMFMQLTGTELT   172
                       ************************************************************

TphC_Comamonas_E6      VIPYKGTAPALIDLIGGNVDVFFDNISSSATYHQAGKVRILAVADEQRSQILPQVPTFAE   240
rTphC_E6               VIPYKGTAPALIDLIGGNVDVFFDNISSSATYHQAGKVRILAVADEQRSQILPQVPTFAE   232
                       ************************************************************

TphC_Comamonas_E6      QQWPAMQAVTFFSVVAPPGTSAEIAQKLQKQMALALSSNDIRKHFQEQGAVPCGWDPSKT   300
rTphC_E6               QQWPAMQAVTFFSVVAPPGTSAEIAQKLQKQMALALSSNDIRKHFQEQGAVPCGWDPSKT   292
                       ************************************************************

TphC_Comamonas_E6      AQFIRQETEKWKKVLKAANVKL   322
rTphC_E6               AQFIRQETEKWKKVLKAANVKL   314
                       **********************
```

Supplementary Fig. 1A. Alignment of native and recombinant TphC sequences. Native TphC from *Comamonas* sp. strain E6 is shown along with the predicted TAT signal peptide M1-S28 (underlined, cut site at S28|N29. NB: To date most SBP have been found to contain Sec rather than TAT-dependent signal peptide. Recombinant TphC with the N-terminal signal peptide sequence removed and replaced with the N-terminal His-6 tag, calculated MW 34kDa.

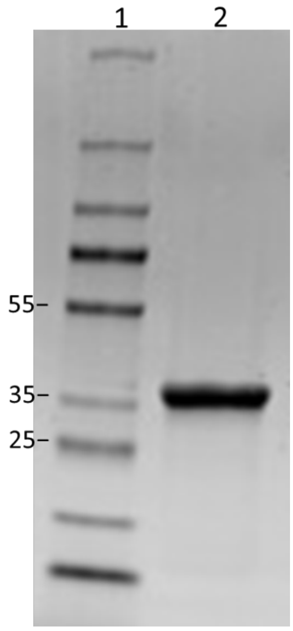

Supplementary Fig. 1B. Purity of TphC sample after gel-filtration chromatography using Superdex-200 26/600 GL column. Representative SDS-PAGE purity of TphC after two consecutive steps of Ni-NTA chromatography and gel filtration chromatography (lane 2)(similar results were obtained for different batches of protein production (n>5). Lane 1 shows a mixture of broad range (11–245 kDa) molecular markers (Prestained Protein Standards, NEB).

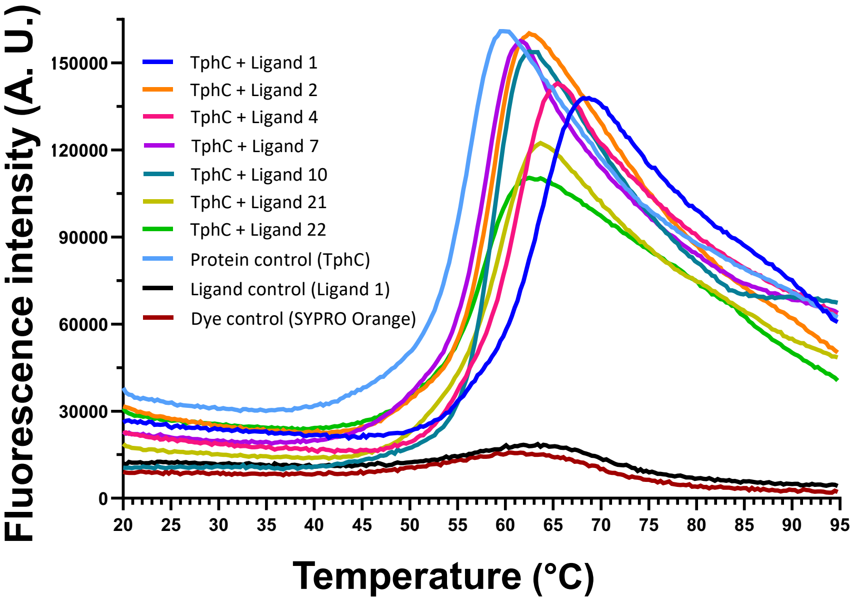

Supplementary Fig. 2. Differential scanning fluorimetry of TphC. Each well of a 96-well plate contained 50 µl of total reaction buffer (25 mM Tris-HCl (pH 7.5)/ 200 mM NaCl), 60 µM of TphC, 1200 µM of ligand and 1x SYPRO orange dye, and fluorescence was monitored at each 1 °C rise in temperature from 20 °C to 95 °C. All experiments were performed in triplicate (*n* = 3) from independent experiments. Shown are only the single raw traces of TphC denaturation for the ligands screened having a stabilising binding interaction with TphC.

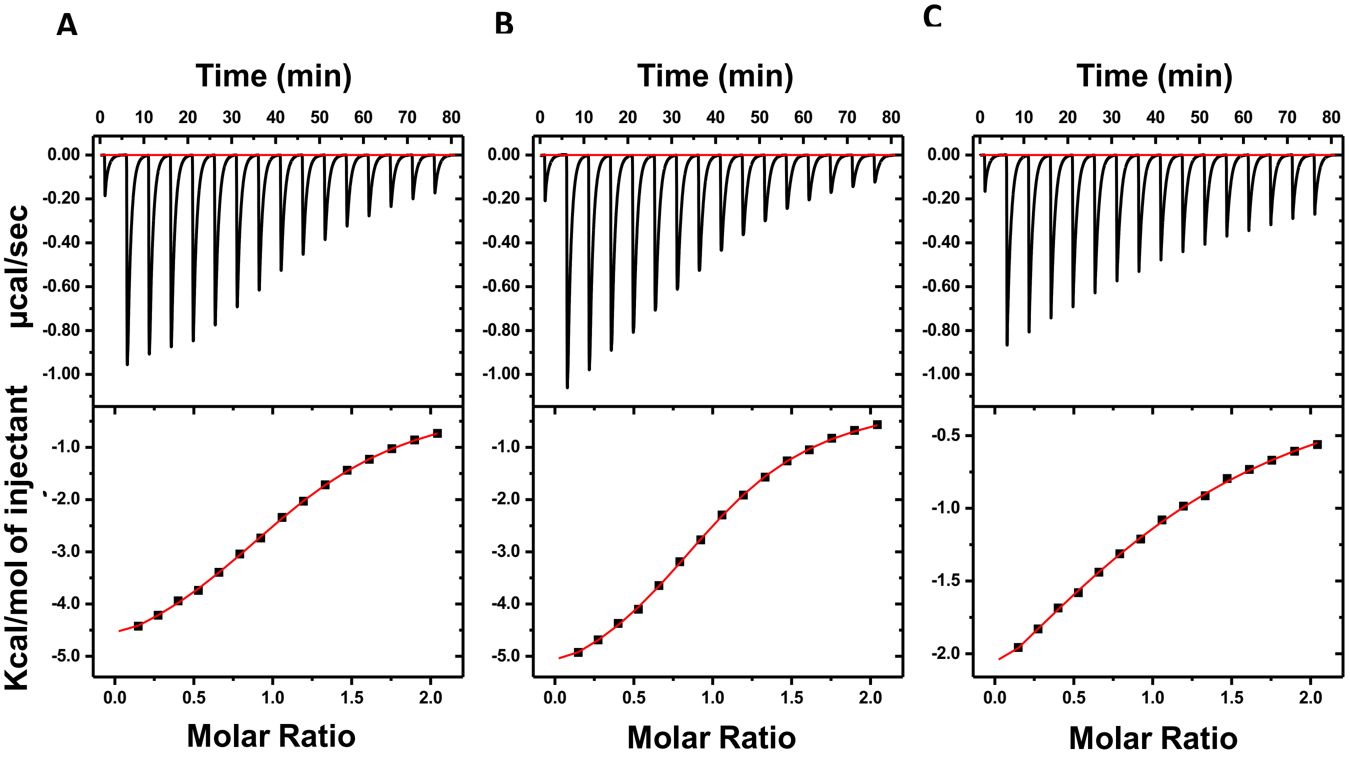

Supplementary Fig. 3. Isothermal titration calorimetry of ligand-hits with TphC. To ascertain the binding interaction of ligand-hits and to determine the corresponding thermodynamic parameters for the interactions between the ligands and TphC, isothermal titration calorimetry was employed. **(A)** 2-aminoterephthalate disodium (**7**) against 500 µM TphC, **(B)** 2,5-pyridinedicarboxylate disodium (**2**) against 500 µM TphC, and **(C)** 2,6-naphthalenedicarboxylate disodium (**22**) against 1000 µM TphC. Experiments were performed at 22 °C at a fixed protein to ligand ratio of 1:10 in Tris-HCl buffer (25 mM Tris-HCl, pH 7.5/200 mM NaCl), with 2.5 µL ligand injections with 300s interval between each injection. Corrected heat rates are shown in the top panel and normalised fit to the data in the bottom panel.

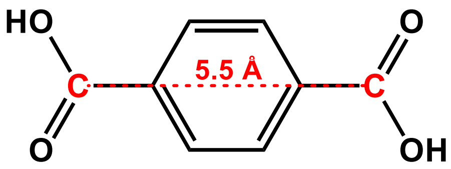

1

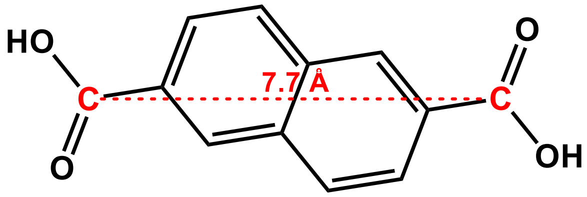

22

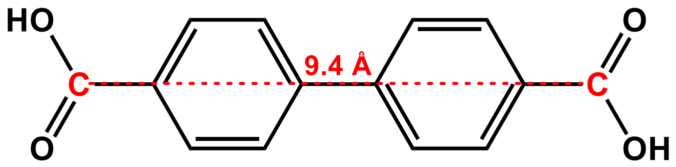

21

Supplementary Fig. 4. Calculated distance between dicarboxylates. Terephthalic acid **1,** 2,6-Naphthalenedicarboxylic acid **22** biphenyl-4,4'-diarboxylic acid **21**.

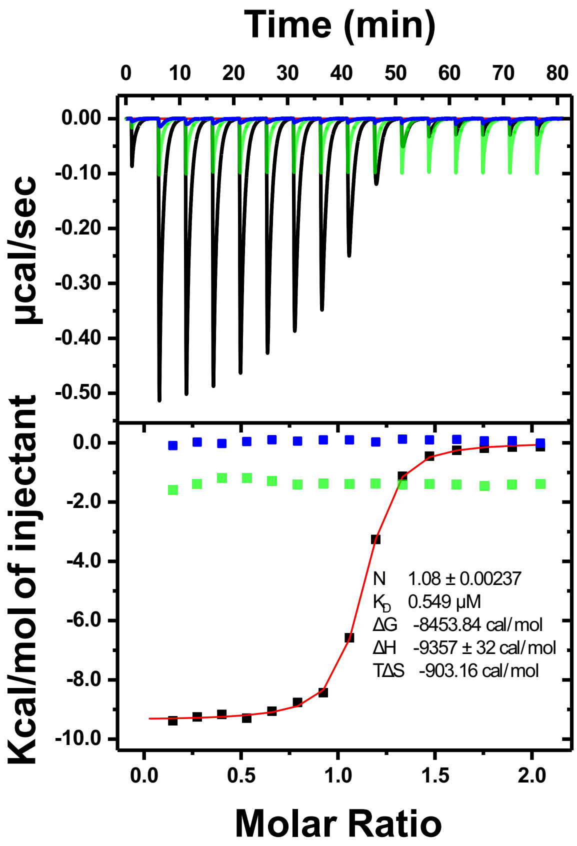

Supplementary Fig. 5. Competitive interaction of biphenyl-4,4′-dicarboxylate and terephthalate for TphC. To ascertain the binding interaction of individual ligand-hits and to determine the corresponding thermodynamic parameters for the competitive interaction between the ligands and TphC, isothermal titration calorimetry was employed. Titration of TphC (100 µM) with terephthalate (**1**) (1000 µM) in the absence of biphenyl-4,4′-dicarboxylate (**21**) (closed black squares), and with TphC saturated with 1000 µM biphenyl-4,4′-dicarboxylate (**21**) (closed blue squares). Titration of TphC (100 µM) with biphenyl-4,4′-dicarboxylate (**21**) (1000 µM) (closed green squares). Experiments were performed at 22 °C with a fixed protein to ligand ratio of 1:10 in Tris-HCl buffer (25 mM Tris-HCl, pH 7.5/200 mM NaCl), with 2.5 µL ligand injections with 300s interval between each injection. Corrected heat rates are shown in the top panel and normalised fit to the data in the bottom panel.

A

```
                                              β1          α1
rTphC        --MGSSHHHHHHGSGENLY------FQSNQPLKIVVPFSAGGTADVLPRLVAEKIRADYA   52
rTctC_4X9T   MHHHHHHSSGVDLGTENLYFQSMQATYPSRPIELIVPYPAGGGTDVLGRAFALASVKHLP   60
rBug27_2QPQ  -----------------ATG------DFPNKPLDIIVTFPPGGGTDMLARLIGNYLTESLG   38
                                         .:*:.::* :  ** :*:* * ..

                 β2          α2            β3
rTphC        GGVIIENKPGAGGNIGADLVFRAPPDGMTVLASPPGPIAINHNLYQKLSFDPTRWV-PVT   111
rTctC_4X9T   QNLIVVNKPGASGAIGWADVINGKPEGYKVALLATDLMT-QPNM--GLTKITHEDFIPIA   117
rBug27_2QPQ  QTAVVENRPGASGNVGARLVADRAPDGYSLLMVN-SSFAVNPGVFRNLPFDPKKDFAAVI   97
                :: *:***.* :*   *    *:* .:     . :: : .:   *     . .  :

                  β4                 α3          β5             α4
rTphC        ILATVPNVLVINPKLPVKSLGEFIAYAKANPKKVTVATQGDGSTSHLTAAMFMQLTGTEL   171
rTctC_4X9T   RLNYDPAAITVRADAPWNTVEEFLAAAKQGDF--RVGNGGNGSTWHLAAAAVEDKTGVKF   175
rBug27_2QPQ  NVAYVPSVFVVPAGSKYKTLGELMAAAKQTNTQVTYGSCGNGTPQHLAGELLNVSAKTHM   157
              :   * .:.:      ::: *::* **        .. *:*:  **:.  .   : ..:

              β6          α5          β7    α6          β8
rTphC        TVIPYKGTAPALIDLIGGNVDVFFDNISSSATYHQAGKVRILAVADEQRSQILPQVPTFA   231
rTctC_4X9T   NHIPFAGAAPAALSLLGGHIEAITVSAAEVYAYTSTGKLKTLAVMSEQRIKGFEKVPTLK   235
rBug27_2QPQ  VHVPYKGCGPALNDVLGSQIGLAVVTASSAIPFIKAGKLQALAVTSKERSALLPEVPTVA   217
               :*: * .**  .::*.::     . :.   : .:**:: *** .::*   : :***.

                          β9            α7                 α8
rTphC        EQQWPAMQAVTFFSVVAPPGTSAEIAQKLQKQMALALSSNDIRKHFQEQGAVPCGWDPSK   291
rTctC_4X9T   ERNI-DISIGTWRGLAVTKGTPPEIVNVLRAATAKIVTEQSLRDALDRQNMGYAYAEGEA   294
rBug27_2QPQ  EQGVAGYELNQWHGLLVPGATPMAVRQKLYDGIAKVMQRDDVQKKLADLGYSTASDGPEV   277
             *:     .   : .: .  .*   : : *    *  :  :.::. :   .   .    .

                      α9
rTphC        TAQFIRQETEKWKKVLKAANVKL-  314
rTctC_4X9T   FGAVMARDHAFYKGLINKLGLKQ-  317
rBug27_2QPQ  FQKMVETDIDRFSALTKQIGLKVD  301
                .:  :   :. : :  .:*
```

B

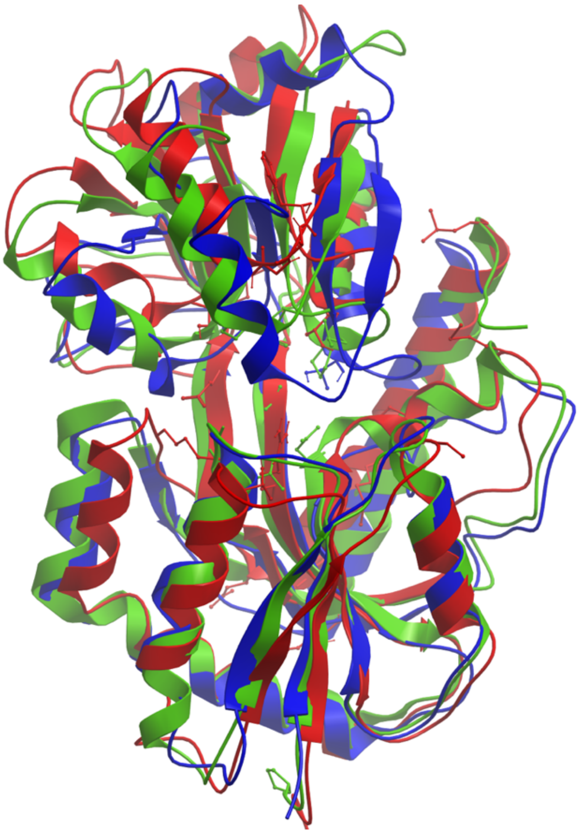

Supplementary Fig. 6. Sequence alignment and overlay of open apo-SBP TTT structures. (A) Sequence alignment of TphC with apo-TctC (PDB 4X9T) and Bug27 (PDB 2QPQ), the percent identity between TphC, TctC and Bug 27, is 27.3% and 30.8% respectively. α-helices indicated in red, β-sheets in green, and the conserved motif between β1-α1 is underlined. **(B)** Overlay of TphC (blue) and TctC (red) Bug27 (green).

A

```
rTphC_E6        ---------MGSSHHHHHHGSGENLYFQ---------SNQPLKIVVPFSAGGTADVLPRL 42
rAdpC_5OEI      MGGSHHHHHHGMASMTGGQQMGRDLYDDDDKDPSSDWPTRQVTLVVPFTSGGTTDMLARL 60
rBugE_2DVZ      ---------------------MRGSHHHHHHGSADAYPSKAIRVIVPFAPGGSTDIIARL 39
rBugD_2F5X      -----------------------MRGSHHHHHHGSEYPERPVNXVVPFAAGGPTDNVARS 37
rMatC_6HKE      ------------------------MASEPDRLDQIDFPVRTVTVVVPFAKGGPTDTVARL 36
                                           .           : :  :***: ** :* : *

rTphC_E6        VAEKIRADYAGGVIIENKPGAGGNIGADLVFRAPPDGMTVLASPPGPIAINHNL-YQKLS 101
rAdpC_5OEI      IAARLSEHYGQSFVIDNRSGESGNIAASYVAKVPADGYTFIIGTPGIHATNRLV-YRTMG 119
rBugE_2DVZ      VTQRMSQELGQPMVVENKGGAGGAIGASEAARAEPDGYTLSIATVSTMAVNPACRPKDLP 99
rBugD_2F5X      LAESXRPTLGETVVVENKGGAGGTIGTTQVARAQPDGYSILLXHAGFST-APSL-YKNPG 95
rMatC_6HKE      ITAEMAKTLGQPIEIENMLGAGGTLAATRVAHAAPDGHTLIVGHLGTHGAAVAL-FPKLA 95
                ::       .  . ::*  * .* :.:  . :.  ** :.     .

rTphC_E6        FDPT-RWVPVTILATVPNVLVINPKLPVKSLGEFIAYAKANPKKVTVATQGDGSTSHLTA 160
rAdpC_5OEI      YDPATDFTPVIVIARVPNLLSVTKSLPVTSVADLISYARQRPRELFYGVSALGSTGHLST 179
rBugE_2DVZ      YDPIKDFQPVTNFANTANVVAVNPKFPAKDFKGFLEELKKNPGKYSYGSSGTCGVLHLMG 159
rBugD_2F5X      YEPYTSFEPIGLVVDVPXTIIARGDFPPNNIKELAEYVKKNADKISLANAGIGAASHLCG 155
rMatC_6HKE      YRPDKDFTPVALLTEMPVLLLARKQFPPKDLSEFASYVESHTDNLNVAHAGFGSVSYASC 155
                : *   : *:  ..     :    .:* ...  :    . .  :   .  .  .. :

rTphC_E6        AMFMQLTGTELTVIPYKGTAPALIDLIGGNVDVFFDNISSSATYHQAGKVRILAVADEQR 220
rAdpC_5OEI      ELFKTMTGVEITAVPYKGSAPMLRDLAEGRVHLTIDNLPASKPLLEAGEIRPLAVTTAKR 239
rBugE_2DVZ      ESFKMATGTDIVHVPYKGSGPAVADAVGGQIELIFDNLPSSMPQIQAGKLRAMAIAWPTR 219
rBugD_2F5X      TXLVEALGVNLLTIPYKGTAPAXNDLLGKQVDLXCDQTTNTTQQITSGKVKAYAVTSLKR 215
rMatC_6HKE      LLLNRLLKVDPTGVPFSGTGPALQALVEGQVDYMCDQIVNAVPALREGKVKAYVIAASER 215
                  :     .:   :*:.*:.*        .:.   *:   :      *:::  .::   *

rTphC_E6        SQILPQVPTFAEQQWPAMQAVTFFSVVAPPGTSAEIAQKLQKQMALALSSNDIRKHFQEQ 280
rAdpC_5OEI      WPPLSHLPTIAEAGVPGYETASWFTVGAPRGTPTEIVTSLNTTVAAFLGSDSGTVKLREI 299
rBugE_2DVZ      IDAIKDVPTFADAGFPVLNQPVWYGLLAPKGTPMDVVNKLRDAAVVALKDPKVIKALDDQ 279
rBugD_2F5X      VPTLPDLPTXDESGYKGFEVGIWHGXWAPKGTPKPVVDKLVKSLQAGLADPKFQERXKQL 275
rMatC_6HKE      DPVVPDVPTAREAGLPGFQVGAWTGLFAPRGTPEPIVAKLNAAVSRALDQSDVRTRLTDL 275
                   : .:**  :      :   :    ** **   :. .*       * . .      :

rTphC_E6        GAVP---CGWDPSKTAQFIRQETEKWKKVLKAANVKL------ 314
rAdpC_5OEI      GAEP---GGGSPQDMQRHVEAEIARWEKVAKTAGIAPL----- 334
rBugE_2DVZ      GSAP---SGNTPEEFAKEIKEQYDWAQDVVKKQNIKLD----- 314
rBugD_2F5X      GAEVLTNEA-NPEALQAKVKQQVPQWAELFKKAGVEKQ----- 312
rMatC_6HKE      GALVPRPEQRAPVVLAQLVQEEISRWEDVVEGTTPLEHHHHHH 318
                *:         *      :. :     .: :
```

B

1:	rTphC_E6	100.00	35.67	33.77	31.21	30.10
2:	rAdpC_5OEI	35.67	100.00	31.63	29.13	33.23
3:	rBugE_2DVZ	33.77	31.63	100.00	33.33	31.94
4:	rBugD_2F5X	31.21	29.13	33.33	100.00	36.98
5:	rMatC_6HKE	30.10	33.23	31.94	36.98	100.00

Supplementary Fig. 7. Sequence alignment and identity matrix scores for the closed holo-SBP TTT structures. (A) Sequence alignment of TphC-TPA with AdpC (PDB 5OEI), BugE (PDB 2DVZ), BugD (PDB 2F5X), and MatC (PDB 6HKE). α-helices indicated in red, β-sheets in green, and the amino acid involved in TPA recognition are in bold and underlined. **(B)** Percent Identity Matrix.

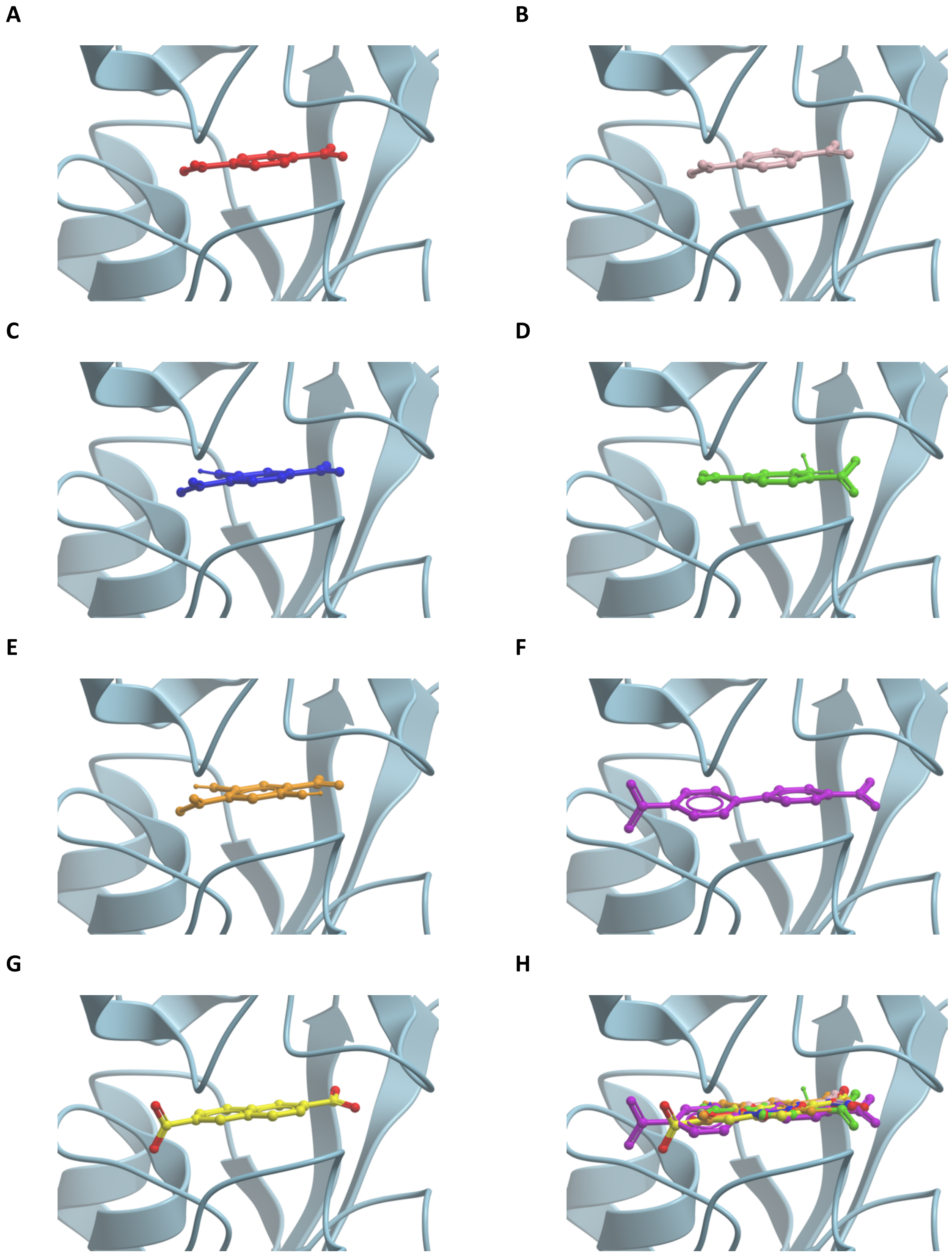

Supplementary Fig. 8. Docked structures. (A) Terephthalate **1**, **(B)** 2,5-Pyridinedicarboxylate **2, (C)** 2-Hydroxyterephthalate **4**, **(D)** 2-aminoyterephthalate **7**, **(E)** 2,5-dihydroxyterephthalate **10**, **(F)** biphenyl-4,4′-dicarboxylate **21**, **(G)** 2,6-naphthalenedicarboxylate **22** **(H)** All ligands overlayed**.** Performed using AutodockVina, all ligands docked well into the closed Tphc structure, with the exception of **22** which required additional pre-energy minimisation (see Methods).

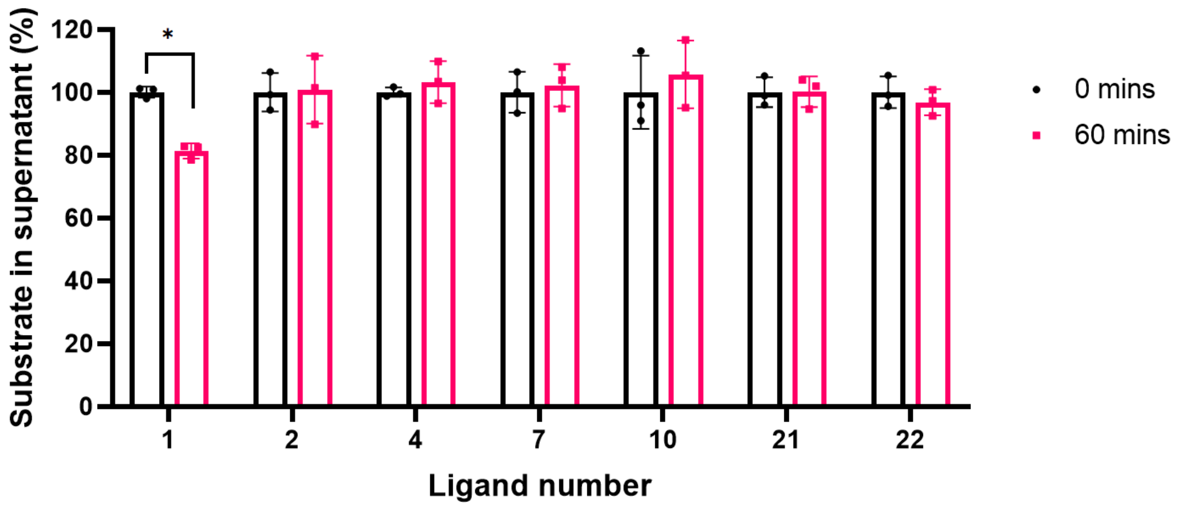

Supplementary Fig. 9. Ligand depletion assays by resting cells of *P. putida* KT2440 ΔpcaGH carrying tphC/tpiAB and the tphAB$_{II}$ operon. Resting cells of *P. putida* KT2440 ΔpcaGH (OD_{600} = 30) transformed with plasmid pJCBAtG were incubated at 30°C with 1 mM of the selected ligand (corresponding to initial substrate percentage). Supernatant ligand concentrations were measured by HPLC at time 0 and after 60 minutes. Results are given as the mean n = 3 with +/- SD. Statistical testing performed with one way ANOVA using Dunnett's multiple comparisons test. '*' indicates a significant p-value of 0.0028.

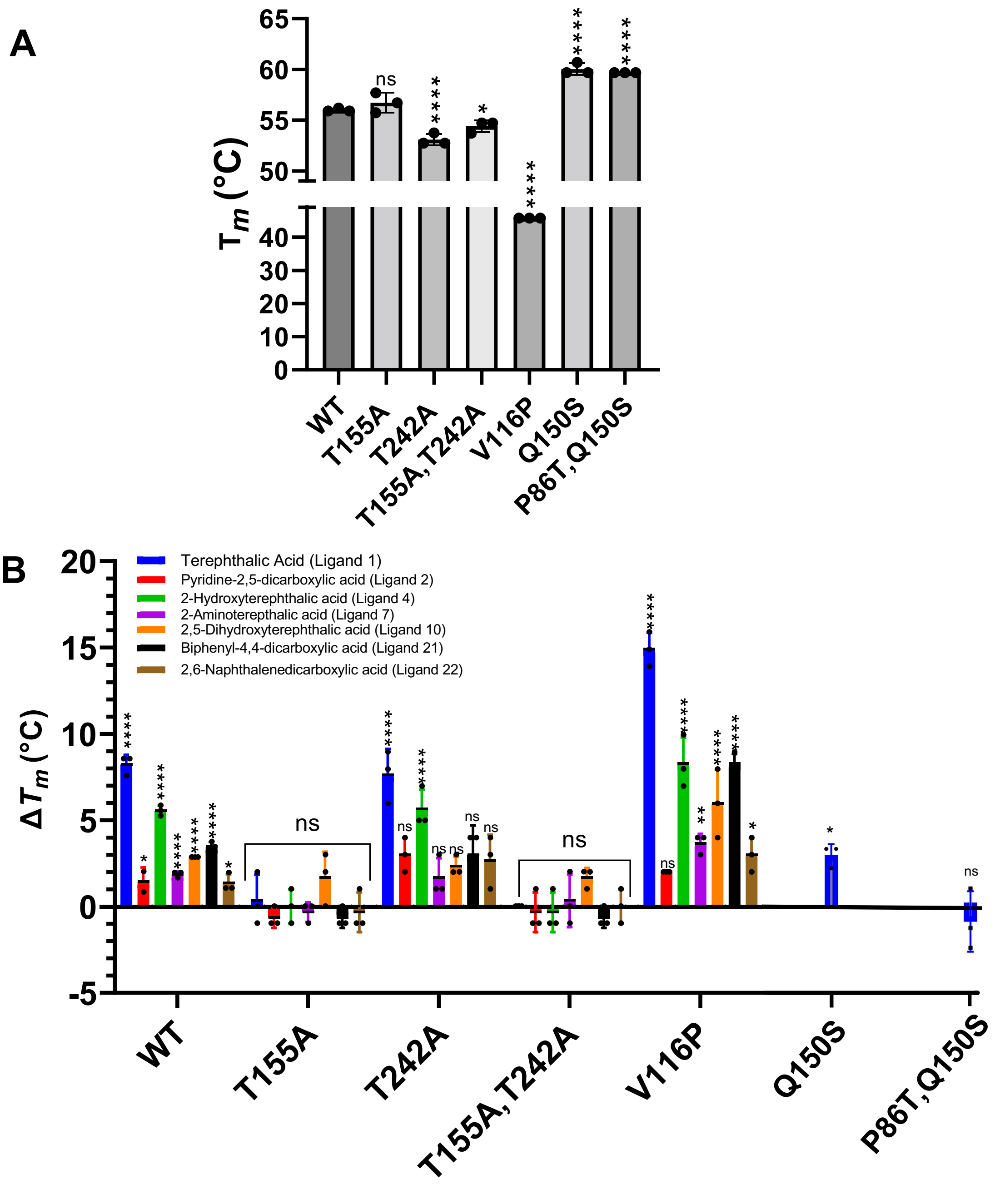

Supplementary Fig. 10. Mutational analysis of amino acids involved in ligand binding (A) Comparison of melting temperature (T_m) for TphC and mutated proteins. Statistical testing performed with one way ANOVA using Dunnett's multiple comparisons test. Noted P values: T242A = <0.0001, T155A,T242A = 0.0122, V116P = <0.0001, Q150S = <0.0001 and P86T,Q150S = <0.0001. (**B**) Comparison of thermal shift of TphC and mutants in presence of TPA analogues. Statistical testing performed with one way ANOVA using Dunnetts multiple comparisons test comparing against the TphC apo control, (*p*-value <0.05), where asterisks denote statistically significant difference (*p* <0.02*; *p* <0.0001****) compared to the control. Noted P values WT (Ligands 1,2,4,7,10,21,22 respectively): <0.0001, 0.0415, <0.0001, <0.0001, <0.0001, <0.0001, 0.0276; T155A: ns; T242A: <0.0001, ns, 0.0003, ns, ns, ns, ns; T155A,T242A: ns; V116P: <0.0001, ns, <0.0001, 0.0051, <0.0001, <0.0001, 0.0243; Q150S (ligand 1

only): 0.0303; P86T,Q150S (ligand 1 only): ns. Each well of a 96-well plate contained 50 µl of total reaction buffer (25 mM Tris-HCl (pH 7.5)/ 200 mM NaCl), 60 µM of TphC, 1200 µM of ligand (as required) and 1x SYPRO orange dye, and fluorescence was monitored at each 1 °C rise in temperature from 20 °C to 95 °C. The data shown are the mean of independent experiments (*n* = 3) and the error bars show the standard deviation (SD) of the mean.

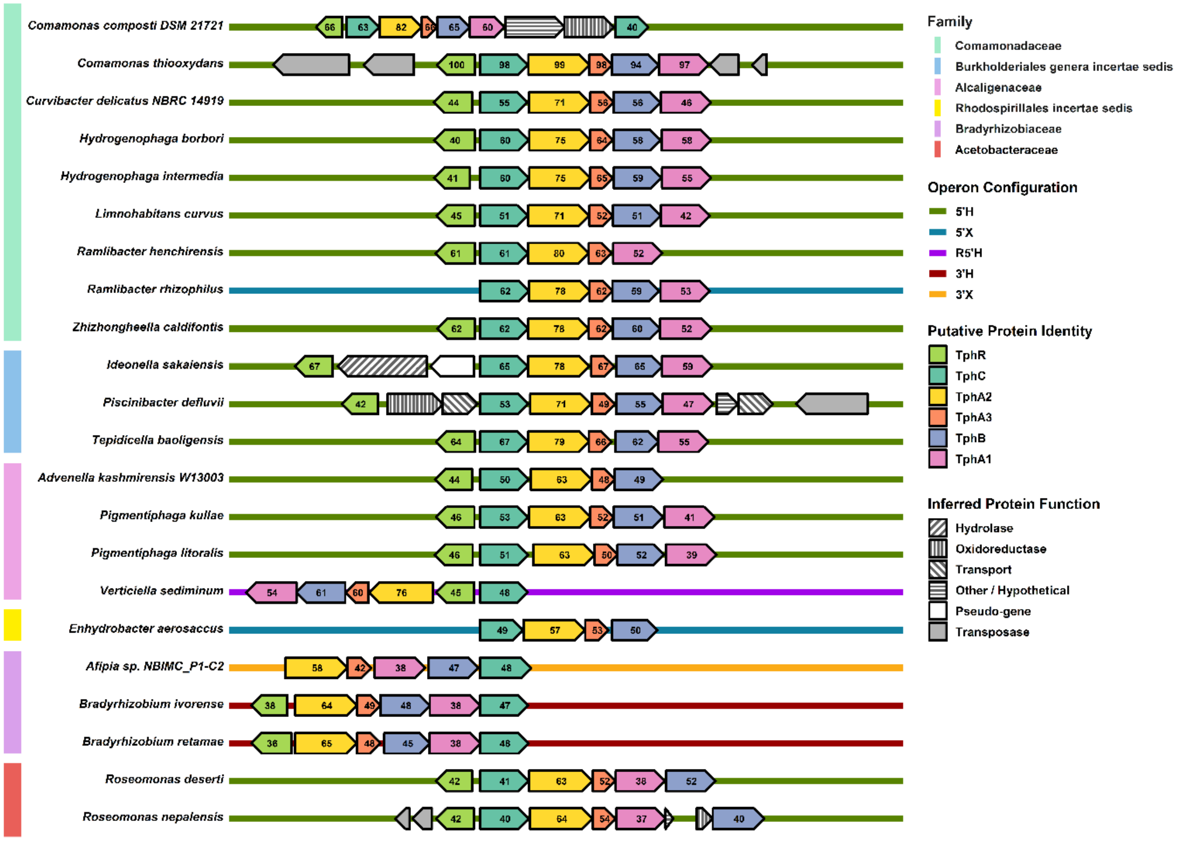

Supplementary Fig. 11. Schematic diagram of operon configuration of all putative *tph*-like operons. Schematic diagram of the operon configuration of all the putative *tph*-like operons. Taxonomic family is shown by coloured boxes to the left of the operon. The values inside the genes indicate the percentage similarity of the encoded protein to the corresponding protein in *Comamonas* sp. strain E6. The operon configuration indicates the location of the genes encoding TphR and TphC relative to the correspsonding intergenic/promoter region. Predominately *tphC* is located at the 5′ end of the operon and *tphR* located head-to-head with respect to the corresponding promoter (5′H). The encoded proteins homologous to those from *Comamonas* sp. strain E6 were putatively assigned as being Tph proteins are shown (putative protein identity). Non-consensus encoded proteins associated with the genomic loci were catagorised using blast2GO are shown (inferred protein function).

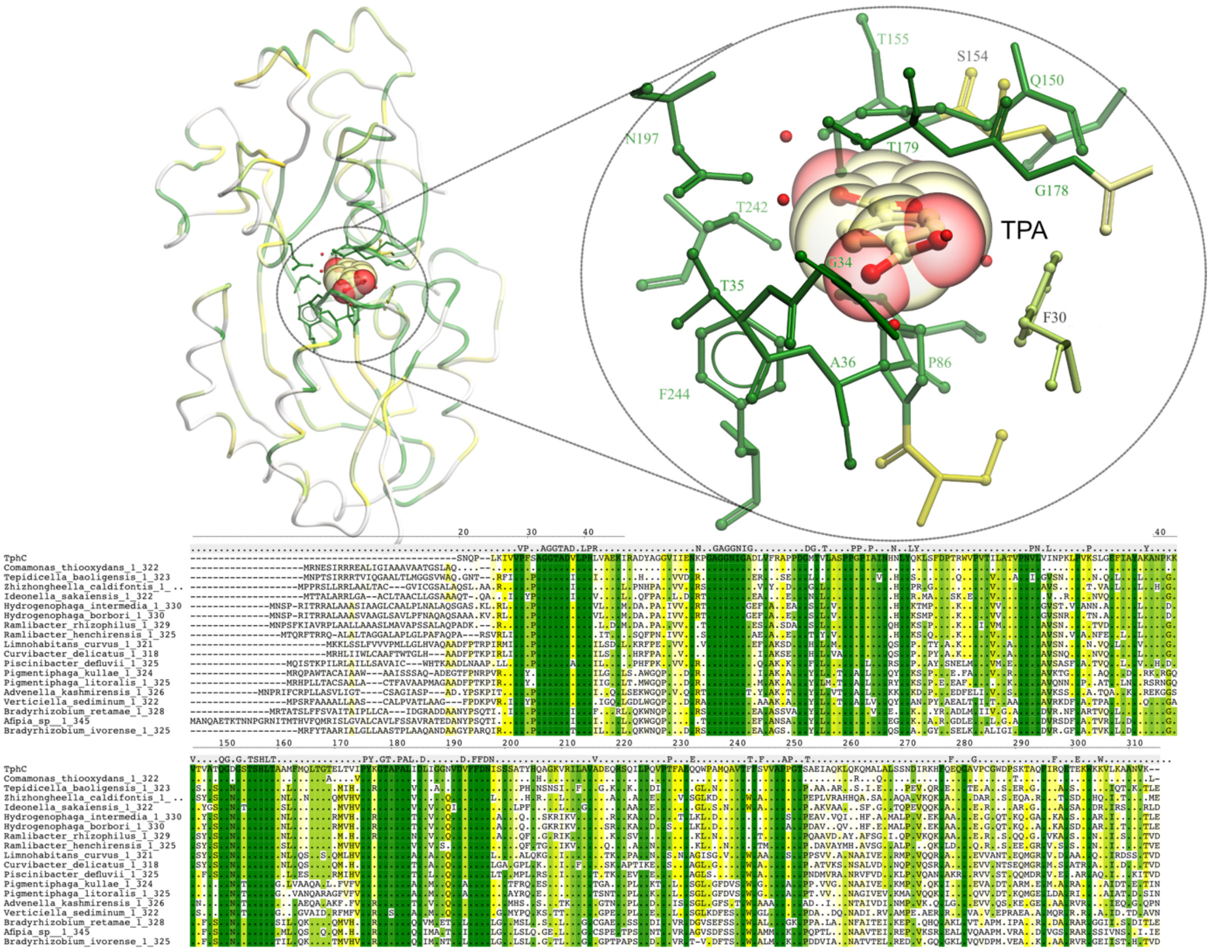

Supplementary Fig. 12. TphC homology alignment. Crystal structure of TphC-TPA complex coloured by sequence conservation (conservation graded from green to yellow). The protein backbone is displayed in protein worm representation and residues involved in the TPA binding site are shown in ball and stick representation and coloured by their respective levels of conservation. TPA is shown in ball and stick and CPK sphere representation and coloured in all atom colours. Water molecules are shown as red spheres. TphC shows a broad distribution of conservation with the binding site and domain contacts being very highly conserved across the 19 sequence alignment. The sequence alignment is coloured by conservation, consensus sequence is shown along with highlighting residue differences across the aligned sequences.

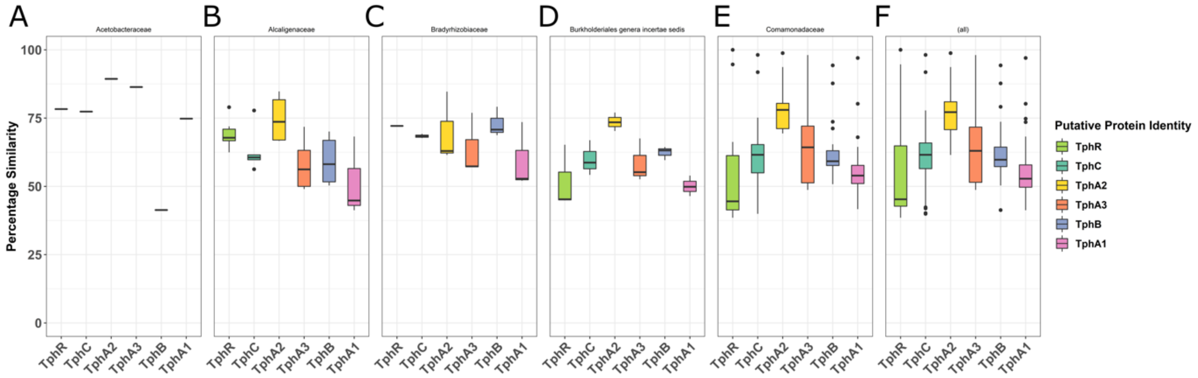

Supplementary Fig. 13. Sequence diversity of the putative *tph* operons. A percent identity matrix (PIM) was calculated for the putative Tph proteins and the Tph proteins from *Comamonas* sp. strain E6. (**A**) PIM comparisons for proteins encoded by operons belonging to taxonomic family *Acetobacteraceae*. (**B**) PIM comparisons for proteins encoded by operons belonging to taxonomic family *Alcaligenaceae*. (**C**) PIM comparisons for proteins encoded by operons belonging to taxonomic family *Bradyrhizobiaceae*. (**D**) PIM comparisons for proteins encoded by operons belonging to taxonomic family *Burkholderiales genera incertae sedis*. (**E**) PIM comparisons for proteins encoded by operons belonging to taxonomic family *Comamonadaceae*. (**F**) PIM comparisons regardless of taxonomic family. Self-identity comparisons and duplicated comparisons were removed from A-F. The percent identity comparisons within each of the groups are shown as boxplots. The upper hinge of the boxplot indicates the third quartile, the middle bar indicates the median and the lower bar indicates the first quartile. The whiskers of the boxplot show the 'maximum' (third quartile + 1.5*the interquartile range) and 'minimum' (first quartile -1.5*the interquartile range) values. All values outside of this range are outliers and are shown as black points.

Supplementary Table 1. Salts synthesised for terephthalate structural analogues.

Ligand	CAS No.	Code*	Salt formula	Formula wt. (g/mol)	Purity (%)
Tetrabromoterephthalic acid	5411-70-1	**9**	$C_8O_4Br_4Na_2$	525.68	59.4
2-Hydroxyterephthalic acid	636-94-2	**4**	$C_8H_4O_5Na_2$	226.09	96.8
2,5-Dihydroxyterephthalic acid	610-92-4	**10**	$C_8H_4O_6Na_2$	242.09	97.5
2-Bromoterephthalic acid	586-35-6	**5**	$C_8H_3O_4BrNa_2$	288.99	81.3
2-Iodoterephthalic acid	1829-22-7	**6**	$C_8H_3O_4INa_2$	335.99	85.6
Biphenyl-4,4'-dicarboxylic acid	787-70-2	**21**	$C_{14}H_8O_4Na_2$	286.16	96.4
2-Aminoterephthalic acid	10312-55-7	**7**	$C_8H_5N_1O_4Na_2$	225.11	88.7
Furan-2,3-dicarboxylic acid	4282-24-0	**14**	$C_6H_2O_5Na_2$	200.06	63.6
Furan-2,5-dicarboxylic acid	3238-40-2	**15**	$C_6H_2O_5Na_2$	200.06	70.3
Pyridine-2,3-dicarboxyic acid	89-00-9	**16**	$C_7H_3N_1O_4Na_2$	211.08	84.8
Pyrazine-2,5-dicarboxylic acid	205692-63-3	**3**	$C_6H_2N_2O_4Na_2$	212.07	97.9
Pyridine-3,4-dicarboxylic acid	490-11-9	**17**	$C_7H_3N_1O_4Na_2$	211.08	86.9
Pyridazine-4,5-dicarboxylic acid	59648-14-5	**18**	$C_6H_2N_2O_4Na_2$	212.07	89.9
Pyrazine-2,3-dicarboxylic acid	89-01-1	**19**	$C_6H_2N_2O_4Na_2$	212.07	ND
Pyridine-2,5-dicarboxlyic acid	100-26-5	**2**	$C_7H_3N_1O_4Na_2$	211.08	97.2
Isophthalic acid	121-91-5	**12**	$C_8H_4O_4Na_2$	210.10	94.6
2-Nitroterephthalic acid	610-29-7	**8**	$C_8H_3N_1O_6Na_2$	255.09	91.6
2,6-Naphthalenedicarboxylic acid	1141-38-4	**22**	$C_{12}H_6O_4Na_2$	260.16	89.6
1,4-Naphthalenedicarboxylic acid	605-70-9	**23**	$C_{12}H_6O_4Na_2$	260.16	88.0

*Number assigned during ligand screening using differential scanning fluorimetry assay.
ND: Not determined.

Supplementary Table 2. Ligands investigated for thermal-shift of TphC using DSF assay.

Code*	Description	Code*	Description
**TphC**	Ligand-free TphC	**31**	Indole-3-acetamide
**1**	Terephthalate Disodium	**32**	4-Hydroxybenzenesulfonamide
**2**	2,5-Pyridinedicarboxylate Disodium	**33**	mono (2-Hydroxyethyl) terephthalate (MHET)
**3**	2,5-Pyrazinedicarboxylate Disodium	**34**	4-(Hydroxymethyl)benzoic acid
**4**	2-Hydroxyterephthalate Disodium	**35**	mono-Methyl terephthalate
**5**	2-Bromoterephthalate Disodium	**36**	Protocatechuic acid
**6**	2-Iodoterephthalate Disodium	**37**	Orotic acid potassium salt
**7**	2-Aminoterephthalate Disodium	**38**	4-Formylbenzoic acid
**8**	2-Nitroterephthalic acid	**39**	4-Formyl-3-hydroxybenzoic acid
**9**	Tetrabromo terephthalate Disodium	**40**	Sodium benzoate
**10**	2,5-Dihydroxyterephthalate Disodium	**41**	3-Methoxybenzoic acid
**11**	Tetrafluoroterephthalic acid	**42**	3-Methoxybenzamide
**12**	Isophthalate Disodium	**43**	4-Methoxybenzoic acid
**13**	Phthalate Sodium	**44**	Gallic acid
**14**	Furan-2,3-dicarboxylate Disodium	**45**	Caffeic acid
**15**	Furan-2,5-dicarboxylate Disodium	**46**	2,4-dihydroxycinnamic acid
**16**	2,3-Pyridinedicarboxylate Disodium	**47**	4-Hydroxyphenylpyruvic acid
**17**	3,4-Pyridinedicarboxylate Disodium	**48**	p-Coumaric acid
**18**	Pyridazine-4,5-dicarboxylate Disodium	**49**	trans-Ferulic acid
**19**	2,3-Pyrazinedicarboxylate Disodium	**50**	4-Nitrocinnamic acid
**20**	Biphenyl-2,2′-dicarboxylic acid	**51**	DL-p-Hydroxyphenyllactic acid
**21**	Biphenyl-4,4′-dicarboxylate Disodium	**52**	Catechol
**22**	2,6-Naphthalenedicarboxylic acid	**53**	bis (2-Hydroxyethyl) terephthalate (BHET)
**23**	1,4-Naphthalenedicaboxylic acid	**54**	Dimethyl terephthalate
**24**	Terephthalamate Sodium	**55**	Methyl 4-(chlorocarbonyl)benzoate
**25**	Potassium 4-nitrophenyl sulfate	**56**	Phenyl acetate
**26**	Phthalamate Sodium	**57**	trans,trans-Muconic acid
**27**	2-Hydroxybenzoic acid (Salicylic acid)	**58**	cis,cis-Muconic acid
**28**	Sulfanilamide	**59**	Adipic acid
**29**	4-Nitrophenylacetonitrile	**60**	Glutaric acid
**30**	4-Nitrotoluene	**61**	Suberic acid

*Number assigned during ligand screening using differential scanning fluorimetry assay.

Supplementary Table 3. X-ray data collection and refinement statistics TphC (open)

	TphC Open
**Wavelength**	0.9762 Å
**Resolution range**	66.49 - 1.97 (2.04 - 1.97)
**Space group**	C 1 2 1
**Unit cell**	141.113 81.2691 186.953 90 109.547 90
**Total reflections**	964530 (98291)
**Unique reflections**	140863 (14055)
**Multiplicity**	6.8 (7.0)
**Completeness (%)**	99.88 (99.70)
**Mean I/sigma(I)**	6.60 (0.89)
**Wilson B-factor**	26.13
**R-merge**	0.2038 (1.372)
**R-meas**	0.2207 (1.484)
**R-pim**	0.08395 (0.5613)
**CC1/2**	0.993 (0.565)
**CC***	0.998 (0.85)
**Reflections used in refinement**	140720 (14014)
**Reflections used for R-free**	7252 (690)
**R-work**	0.1826 (0.2760)
**R-free**	0.2212 (0.3153)
**CC(work)**	0.965 (0.795)
**CC(free)**	0.950 (0.727)
**Number of non-hydrogen atoms**	14975
**macromolecules**	13318
**ligands**	20
**solvent**	1649
**Protein residues**	1757
**RMS(bonds)**	0.013
**RMS(angles)**	0.90
**Ramachandran favored (%)**	97.25
**Ramachandran allowed (%)**	2.69
**Ramachandran outliers (%)**	0.06
**Rotamer outliers (%)**	0.21
**Clashscore**	2.45
**Average B-factor**	29.21
**macromolecules**	28.64
**ligands**	38.86
**solvent**	33.82

Statistics for the highest-resolution shell are shown in parentheses.

Supplementary Table 4. X-ray data collection and refinement statistics for TphC-TPA (closed)

	TphC
**Wavelength**	0.9795 Å
**Resolution range**	46.17 - 2.4 (2.486 - 2.4)
**Space group**	C 2 2 21
**Unit cell**	69.25 150.06 58.57 90 90 90
**Total reflections**	81141 (7006)
**Unique reflections**	12237 (1172)
**Multiplicity**	6.6 (6.0)
**Completeness (%)**	99.25 (97.10)
**Mean I/sigma(I)**	9.37 (1.70)
**Wilson B-factor**	37.00
**R-merge**	0.1682 (1.057)
**R-meas**	0.1825 (1.157)
**R-pim**	0.07006 (0.4617)
**CC1/2**	0.994 (0.505)
**CC***	0.999 (0.819)
**Reflections used in refinement**	12236 (1172)
**Reflections used for R-free**	620 (66)
**R-work**	0.1789 (0.2640)
**R-free**	0.2169 (0.2834)
**CC(work)**	0.960 (0.786)
**CC(free)**	0.954 (0.639)
**Number of non-hydrogen atoms**	2361
**macromolecules**	2219
**ligands**	16
**solvent**	130
**Protein residues**	294
**RMS(bonds)**	0.003
**RMS(angles)**	0.53
**Ramachandran favored (%)**	95.55
**Ramachandran allowed (%)**	4.11
**Ramachandran outliers (%)**	0.34
**Rotamer outliers (%)**	1.26
**Clashscore**	2.67
**Average B-factor**	39.59
**macromolecules**	39.65
**ligands**	28.90
**solvent**	39.59

Statistics for the highest-resolution shell are shown in parentheses.

Supplementary Table 5. Comparison of closed TphC with the TTT-SBP homolog structures available in the PDB.

SBP/Source	Identity (%)	RMSD Dom1*	RMSD Dom2*	Pocket Vol. (Å^3)	PDB Accession	Ref.
Rpa4515-oxoadipate/*R. palustris*	37	0.593	0.507	342	5OEI	[1]
Rpa4515-adipate/*R. palustris*	37	0.597	0.480	311	5OKU	[1]
BugE-glutamate/*Bordetella pertussis*	34	0.583	0.533	178	2DVZ	[2]
Bug27 (apo/open)/*Bordetella pertussis*	29	0.470	0.891	-	2QPQ	[3]
BugD-aspartate/*Bordetella pertussis*	30	0.718	0.569	156	2F5X	[4]
MatC (Rpa3494)-malate/ *Rhodopseudomonas palustris*	33	0.480	0.488	166	6HKE	[5]
TctC (apo/open)/*Polaromonas* sp.	26	0.837	0.446	N/A	4X9T	-

* Domains 1 and 2.

Supplementary Table 6. Docking scores for selected ligands

Ligand	Score (kcal mol^{-1})
Terephthalate **1**	-7.8
2,5-Pyridinedicarboxylate **2**	(-8.0) -7.6[1]
2-Hydroxyterephthalate **4**	-8.3
2-Aminoterephthalate **7**	-7.5
2,5-Dihydroxyterephthalate **10**	-8.3
Biphenyl-4,4′-dicarboxylate **21**	-5.7 (-9.5)[2]
2,6-Naphthalenedicarboxylate **22**	-7.9

[1] For **2** the second lowest-scoring docking pose was selected as the lowest was in a slightly different orientation to that of the other ligands. [2] **21** was not successfully docked, so the docked pose after energy minimising the protein with **21** in the active site was used for illustrative purposes.

Supplementary Table 7. Primers Used for Site Directed Mutagenesis

	Fwd	Rev
P86T	TTAGCTTCTACCCCCGGCCCGA	TCGGGCCGGGGGTAGAAGCTAA
T155A	GAGTCACCTGACTGCCGCGATG	GCTGAGCCATCGCCTTGGGTCGC
V116P	GCCCAACGTGCTGGTAATTAAC	GGCGTCGCTAAAATGGTTACGGG

References

1. Rosa, L. T., Dix, S. R., Rafferty, J. B. & Kelly, D. J. Structural basis for high-affinity adipate binding to AdpC (RPA4515), an orphan periplasmic-binding protein from the tripartite tricarboxylate transporter (TTT) family in Rhodopseudomonas palustris. *FEBS J.* **284**, 4262–4277 (2017).

2. Huvent, I. *et al.* Structural analysis of Bordetella pertussis BugE solute receptor in a bound conformation. *Acta Crystallogr. Sect. D Biol. Crystallogr.* **62**, 1375–1381 (2006).

3. Herrou, J. *et al.* Structure-based Mechanism of Ligand Binding for Periplasmic Solute-binding Protein of the Bug Family. *J. Mol. Biol.* **373**, 954–964 (2007).

4. Huvent, I. *et al.* Crystal structure of Bordetella pertussis BugD solute receptor unveils the basis of ligand binding in a new family of periplasmic binding proteins. *J. Mol. Biol.* **356**, 1014–1026 (2006).

5. Rosa, L. T., Dix, S. R., Rafferty, J. B. & Kelly, D. J. A New Mechanism for High-Affinity Uptake of C4-Dicarboxylates in Bacteria Revealed by the Structure of Rhodopseudomonas palustris MatC (RPA3494), a Periplasmic Binding Protein of the Tripartite Tricarboxylate Transporter (TTT) Family. *J. Mol. Biol.* **431**, 351–367 (2019).
