# Supplementary material for: Structural basis of terephthalate recognition by solute binding protein TphC
Source: Nat Commun. 2021 Oct 29;12:6244. doi: 10.1038/s41467-021-26508-0 (PMC8556258; doi:10.1038/s41467-021-26508-0)
Supplement: Supplementary file 2 — Description of Additional Supplementary Files [file 41467_2021_26508_MOESM2_ESM.pdf]

Description of Additional Supplementary Files

File Name: Supplementary Data 1
Description: Phylogenetic and genomic context analysis of tph proteins and operons (A) Taxonomic information of the TphC homologues. (B) TphC homologues sequence analysis. (C) TphC homologues sequence analysis for SBP belonging to tph-like operons. (D) Genomic context analysis – sequences flanking (+/-7000 nts) the TphC homolog. (E) Percent Identity Matrix of proteins belonging to tph-like operons
